# Proteomic Profiling of the Large‐Vessel Vasculitis Spectrum Identifying Shared Signatures of Innate Immune Activation and Stromal Remodeling

**DOI:** 10.1002/art.43110

**Published:** 2025-02-27

**Authors:** Robert T. Maughan, Erin Macdonald‐Dunlop, Lubna Haroon‐Rashid, Louise Sorensen, Natalie Chaddock, Shauna Masters, Andrew Porter, Marta Peverelli, Charis Pericleous, Andrew Hutchings, James Robinson, Taryn Youngstein, Raashid A. Luqmani, Justin C. Mason, Ann W. Morgan, James E. Peters

**Affiliations:** ^1^ Imperial College London London United Kingdom; ^2^ University of Leeds Leeds United Kingdom; ^3^ University of Leeds and NIHR Leeds Biomedical Research Centre, Leeds Teaching Hospitals NHS Trust Leeds United Kingdom; ^4^ University of Oxford Oxford United Kingdom; ^5^ London School of Hygiene & Tropical Medicine London United Kingdom

## Abstract

**Objective:**

Takayasu arteritis (TAK) and giant cell arteritis (GCA), the most common forms of large‐vessel vasculitis (LVV), can result in serious morbidity. Understanding the molecular basis of LVV should aid in developing better biomarkers and treatments.

**Methods:**

Plasma proteomic profiling of 184 proteins was performed in two cohorts. Cohort 1 included patients with established TAK (n = 96) and large‐vessel GCA (LV‐GCA) (n = 35) in addition to healthy control participants (HCs) (n = 35). Cohort 2 comprised patients presenting acutely with possible cranial GCA (C‐GCA) in whom the diagnosis was subsequently confirmed (C‐GCA, n = 150) or excluded (Not C‐GCA, n = 89). Proteomic findings were compared to published transcriptomic data from LVV‐affected arteries.

**Results:**

In cohort 1, comparison to HCs revealed 52 differentially abundant proteins (DAPs) in TAK and 72 DAPs in LV‐GCA. Within‐case analyses identified 16 and 18 disease activity–associated proteins in TAK and LV‐GCA, respectively. In cohort 2, comparing C‐GCA versus not C‐GCA revealed 31 DAPs. Analysis within C‐GCA cases suggested the presence of distinct endotypes, with more pronounced proteomic changes in the biopsy‐proven subgroup. Cross‐comparison of TAK, LV‐GCA, and biopsy‐proven C‐GCA revealed highly similar plasma proteomic profiles, with 26 shared DAPs including interleukin 6 (IL‐6), monocyte/macrophage‐related proteins (CCL7, CSF1), tissue remodeling proteins (TIMP1, TNC), and novel associations (TNFSF14, IL‐7R). Plasma proteomic findings reflected LVV arterial phenotype; for 42% of DAPs, the corresponding gene was differentially expressed in tissue.

**Conclusion:**

These findings suggest shared pathobiology across the LVV spectrum involving innate immunity, lymphocyte homeostasis, and tissue remodeling. Network‐based analyses highlighted immune‐stromal cross‐talk and identified novel therapeutic targets (eg, TNFSF14).

## INTRODUCTION

Takayasu arteritis (TAK) and giant cell arteritis (GCA), the most common forms of large‐vessel vasculitis (LVV) in adults, are characterized by granulomatous arterial inflammation. Progressive damage to arterial walls typically results in stenotic remodeling with consequent tissue ischemia and manifestations such as sight loss, stroke, myocardial infarction, and limb claudication.^1^ Despite phenotypic similarities, TAK and GCA have different demographics, particularly age at onset, and, to a lesser extent, they affect different arterial territories. TAK affects the aorta and its major branches, whereas traditionally GCA was described as involving the cranial arteries such as the temporal artery (a pattern of disease now referred to as cranial GCA [C‐GCA]). However, following advances in noninvasive vascular imaging techniques, it became clear that the aorta and other large vessels are frequently affected in GCA, and some patients have a large‐vessel‐type presentation (large‐vessel GCA [LV‐GCA]) with nonspecific constitutional symptoms and/or limb claudication similar to TAK.^2^, ^3^ Frequent large‐vessel involvement in GCA and similar histopathologic changes has led to debate regarding whether TAK and GCA represent varying manifestations of the same disease.^4^, ^5^ This question has important implications for drug development and clinical trial design. However, such comparisons are currently limited by an incomplete understanding of the pathogenic underpinnings of these diseases and a lack of comparative molecular data across LVV phenotypes.

There are several important challenges in the clinical management of TAK and GCA. Both initial diagnosis and recognition of relapse may be delayed and are made more difficult by a lack of effective biomarkers.^6^ Blood tests such as C‐reactive protein (CRP) lack specificity, whereas vascular imaging can be insensitive, particularly in glucocorticoid‐treated patients, and impractical for frequent serial monitoring.^7^, ^8^ In the era before widespread access to ultrasound scans (USS), the diagnosis of C‐GCA was confirmed by performing a temporal artery biopsy (TAB). Although considered the “gold standard” diagnostic test, the presence of skip lesions may lead to false‐negative results. Therefore, a negative biopsy does not exclude the diagnosis of GCA. Progress in the treatment of LVV, particularly the development of targeted biologic therapy, lags behind that of other rheumatic diseases. Accordingly, there is an overreliance on long‐term glucocorticoids to maintain disease control with resulting iatrogenic harm.^9^, ^10^ Thus, there is a need for better biomarkers and novel therapeutics to improve patient outcomes.

Proteomic profiling has the potential to address these challenges.^11^ Proteins are the effector molecules of most biologic functions and the targets of most drugs. Given the proximity of arterial tissue to the circulation, blood‐based proteomics is likely to be informative in LVV. Specifically, we hypothesized that the levels of inflammation‐ and cardiovascular‐related proteins would provide a read‐out of disease activity and arterial pathobiology in patients with LVV and enable the evaluation of molecular similarities and differences between GCA and TAK. To this end, we performed proteomic profiling of 184 circulating proteins in two independent cohorts that included 281 patients with TAK, LV‐GCA, or C‐GCA. We identified protein signatures associated with each LVV type and with disease activity. Cross‐disease comparison revealed a striking similarity between the proteomic profiles of active LVV types. Our data indicate the shared dysregulation of innate immune and tissue remodeling pathways and highlight the potential for therapeutics targeting immune‐stromal cross‐talk.

## PATIENTS AND METHODS

### Cohort 1 study participants

Patients with TAK or LV‐GCA were recruited from the Hammersmith Hospital (Imperial College Healthcare NHS Trust, United Kingdom) between 2013 and 2020. Patients with TAK fulfilled EULAR/American College of Rheumatology (ACR) 2022 classification criteria,^12^ and they all had typical patterns of arterial involvement in radiologic assessments. Patients with LV‐GCA were >50 years old at onset with radiologic evidence of LVV, as defined previously.^13^ Three patients with LV‐GCA had concurrent temporal artery involvement (confirmed by temporal artery USS and/or TAB). Healthy control participants (HCs) were recruited locally from hospital and college staff and had no history of inflammatory or cardiovascular disease. Citrate blood samples were centrifuged at 1,000*g* for 10 minutes within four hours of venipuncture, and plasma was stored at −80°C until use. Disease activity was assessed using the Indian Takayasu Clinical Activity Score (ITAS2010) for TAK^14^ and the National Institutes of Health (NIH) score for LV‐GCA.^15^ Active disease was defined by an ITAS2010 score ≥1 or ITAS‐CRP score ≥2 for TAK and by an NIH score ≥2 for LV‐GCA. All inactive cases were retrospectively confirmed to be relapse free for one year following sample collection. For the purpose of further investigation within inactive TAK cases, “durable clinical remission” was defined as the following: (1) absence of all signs, symptoms, and laboratory features attributable to active TAK (as per the EULAR definition^16^); (2) absence of arterial progression on serial vascular imaging; (3) criteria 1 and 2 sustained for the past three years; and (4) successful cessation of all immunosuppressive treatment. Patients and HCs provided written informed consent, and samples were collected as a subcollection registered with the Imperial College Healthcare Tissue Bank (license: 12275; National Research Ethics Service approval 17/WA/0161).

### Cohort 2 study participants

The Temporal Artery Biopsy Versus Ultrasound in Diagnosis of GCA (TABUL) was an international, multicenter, prospective study that compared the sensitivity and specificity of temporal artery ultrasound to biopsy in 381 patients with suspected C‐GCA^17^ (ClinicalTrials.gov identifier: NCT00974883). Reference diagnosis of C‐GCA or Not C‐GCA at six months was based on a combination of baseline signs and symptoms, blood tests, TAB, fulfillment of ACR 1990 GCA classification criteria^18^, clinical course during the follow‐up period, final consultant diagnoses, and verification by an expert review panel, as described previously.^17^

Cranial ischemic complications were defined as permanent ocular or nonocular conditions at presentation. Ocular complications included the following: anterior ischemic optic neuropathy, branch retinal artery occlusion, cilioretinal artery occlusion, cranial nerve palsy (third, fourth, or fifth), central retinal artery occlusion, posterior ischemic optic neuropathy, relative afferent pupillary defect, irreversible visual loss, irreversible visual field defect, irreversible ocular motility or irreversible diplopia. Nonocular cranial complications included the following: scalp necrosis, tongue necrosis, and cerebrovascular accident at presentation considered secondary to GCA. Polymyalgic symptoms at presentation are also reported but do not represent a confirmed diagnosis of polymyalgia rheumatica (PMR).

Citrated blood samples were collected as soon as feasible after starting glucocorticoid treatment (median 2 days, interquartile range [IQR] 1–4 days) and were centrifuged at 2,500*g* for 15 minutes within 1.5 hours of collection, and plasma was stored at −80°C until use. Because of funding constraints and biosample availability, we performed Olink proteomic assays on 239 patient samples of the 381 patients recruited to TABUL. We included all available samples from patients with a diagnosis of C‐GCA. We selected a subset of sex‐ and age‐ (±5 years) matched patients without C‐GCA such that the ratio of C‐GCA to Not C‐GCA was approximately 2:1. As part of the study design, we selected an equal proportion of patients with cranial ischemic complications in the Not C‐GCA group as in the C‐GCA group. Overall study approval was granted by the Berkshire Research Ethics Committee (09/H0505/132), and approval was also granted at local participating clinical sites.

### Proteomic analysis

A total of 184 proteins were measured by proximity extension assay using two Olink Target panels, “Inflammation 1” and “Cardiometabolic,” at the Leeds Immunogenomics Facility, University of Leeds. To provide a succinct and standardized nomenclature, we report proteins by the symbols of the genes encoding them (see Supplementary Data 1 for a full list of proteins and associated full names and accession numbers). Cohort 1 and 2 samples were processed and analyzed independently, but proteomic measurements were performed in the same facility. We designed assay plates such that samples were balanced across plates according to disease grouping and disease activity status, with randomization to determine well position within plates. Proteomic data were normalized using standard Olink workflows, which includes interplate normalization, to produce measures of relative protein abundance (“Normalised Protein eXpression [NPX]”) (log_2_ scale). For visualization, we transformed to Z scores (mean = 0, SD = 1). Because of a technical fault with a PCR machine during the running of one plate on the Inflammation 1 panel for cohort 2, it was necessary to rerun this plate as a separate batch. Principal component analysis (PCA) revealed a batch effect, with samples from this plate separated from samples on the other three plates run as part of the first batch. We therefore adjusted for this batch effect for proteins measured on the Inflammation 1 panel in cohort 2, using batch (a binary variable) as a covariate in linear model based differential abundance analyses. For situations requiring batch‐correction outside of differential abundance testing (eg, visualization of protein levels using violin plots and heatmaps and network analyses), the residuals from the linear model (in Wilkinson notation) NPX ~ batch were used to generate batch‐corrected protein values. Further PCA of these residuals confirmed that the batch effect had been removed. The residuals were then converted to Z scores before visualization or other downstream analyses. Cardiometabolic panels for cohort 1 were not affected by this issue and were analyzed as a single batch.

Proteins with >75% of samples below the lower limit of detection were removed, resulting in 158 and 167 protein measurements for cohort 1 and 2, respectively. Sample‐level quality control (QC) was performed using internal assay controls, boxplots of relative protein abundance values and PCA for outlier detection. In cohort 1, three samples (1 HC sample and 2 TAK samples) were excluded from Inflammation 1 panel measurements because of amplification failures. In cohort 2, 15 samples (9 C‐GCA samples and 6 not C‐GCA samples) were excluded from the Inflammation 1 panel measurements, and 3 samples (all C‐GCA) were excluded from the Cardiometabolic panel measurements because of amplification failures and flagged status in internal QC checks. When possible, all available data were analyzed (eg, differential abundance analyses), but some analyses (eg, hierarchical clustering, PCA, multiple linear regression vs clinical parameters) necessitated using only complete data.

Differential protein abundance was performed using linear models (*lm* function in R). For a given protein, protein abundance was regressed on disease status (encoded as 0 or 1). The beta coefficient for the disease status term represents the estimated log_2_ fold change (log_2_FC) in the protein level between groups under comparison. For example, for the analysis of TAK versus HCs, the regression model was NPX ~ D, in which NPX was log_2_ protein level (continuous variable) and D was a binary variable, encoded as 0 for HC and 1 for TAK. Correction for multiple testing (multiple proteins) was performed using the Benjamini‐Hochberg method (via the *p.adjust* function in R), and an adjusted *P* value of <0.05 (ie, false‐discovery rate <5%) was defined as significant.

### Protein annotation

The 184 proteins measured were highly enriched for pathways related to immune function and the cardiovascular system, as demonstrated by Reactome pathway overrepresentation analysis (Supplementary Data 2). Because of this enrichment, proteins were manually classified as “cytokine related,” “growth factor related,” “chemokine, other immune/inflammatory related protein,” “extracellular matrix related,” or “other function” to facilitate the annotation of differential abundance results. This was done using a combination of public resources including Gene Ontology terms, pathway, and functional databases. The full list of proteins and associated classifications is provided in Supplementary Data 1.

### Network analysis

The protein–protein interaction network between LVV‐associated proteins with directionally concordant changes across LVV types was constructed using high‐confidence interactions (confidence ≤0.9) sourced from the Search Tool for the Retrieval of Interacting Genes/Proteins (STRING).^19^ No additional filtering of interactions was performed. The protein coexpression network was created using interprotein correlation of abundance values. Node edges were defined as Pearson r ≥ 0.6. Cohort 1 (TAK and LV‐GCA) and cohort 2 (C‐GCA) networks were computed individually and then intersected so that only correlations present in both networks feature in the final network. Both networks were plotted using the igraph package in R.^20^

### Tissue gene expression of LVV‐associated proteins

Bulk RNA sequencing (RNA‐seq) data from human tissues were accessed from the Genotype‐Tissue Expression (GTEx) database as median transcripts per million values per tissue.^21^ Data preprocessing included the following: removal of sex‐specific organ data (ie, cervix, breast, vagina, testis, fallopian tubes), removal of purified cell data (eg, cultured fibroblasts), and when there were multiple sample types per tissue group (eg, artery‐coronary or artery‐aorta), the highest expression value was used for that tissue type. Enhanced tissue expression was defined as more than four‐fold higher than the average expression in other tissues, as done previously by the Human Protein Atlas.^22^

### RNA‐seq analysis of LVV arterial tissue

A previous study compared the transcriptomic profile of inflammatory and noninflammatory aortic aneurysms using bulk RNA‐seq.^23^ Gene‐level count data were accessed and filtered for cases of inflammatory aneurysm associated with GCA (n = 8) for comparison with noninflammatory cases (n = 25). Data were normalized, genes with low expression were removed, and groups were compared using the standard edgeR package methodology.^24^ Differentially expressed genes (DEGs) were defined using Benjamini‐Hochberg adjusted *P* < 0.05. DEGs were then compared with LVV‐associated plasma proteins regarding overlap and Pearson correlation of log_2_FC in each disease.

### Supervised learning

Predictive models to develop disease activity biomarkers in TAK and diagnostic markers in C‐GCA were constructed using the *glmnet* ^25^ and *caret* ^26^ R packages. For these analyses, data were split into training and test sets. Modeling fitting was performed on the training set using five‐fold cross‐validation and performance and then evaluated in the test set. Full details are provided in the Supplementary Methods.

### Other analyses and data and code availability

Details of additional analyses are provided in the Supplementary Information File. The post QC proteomic data are available from figshare (https://doi.org/10.6084/m9.figshare.26928211.v1). The raw proteomic data and the R code for QC and differential abundance analysis are available from Github (https://github.com/r-maughan/LVV_Olink).

## RESULTS

**Figure 1:**
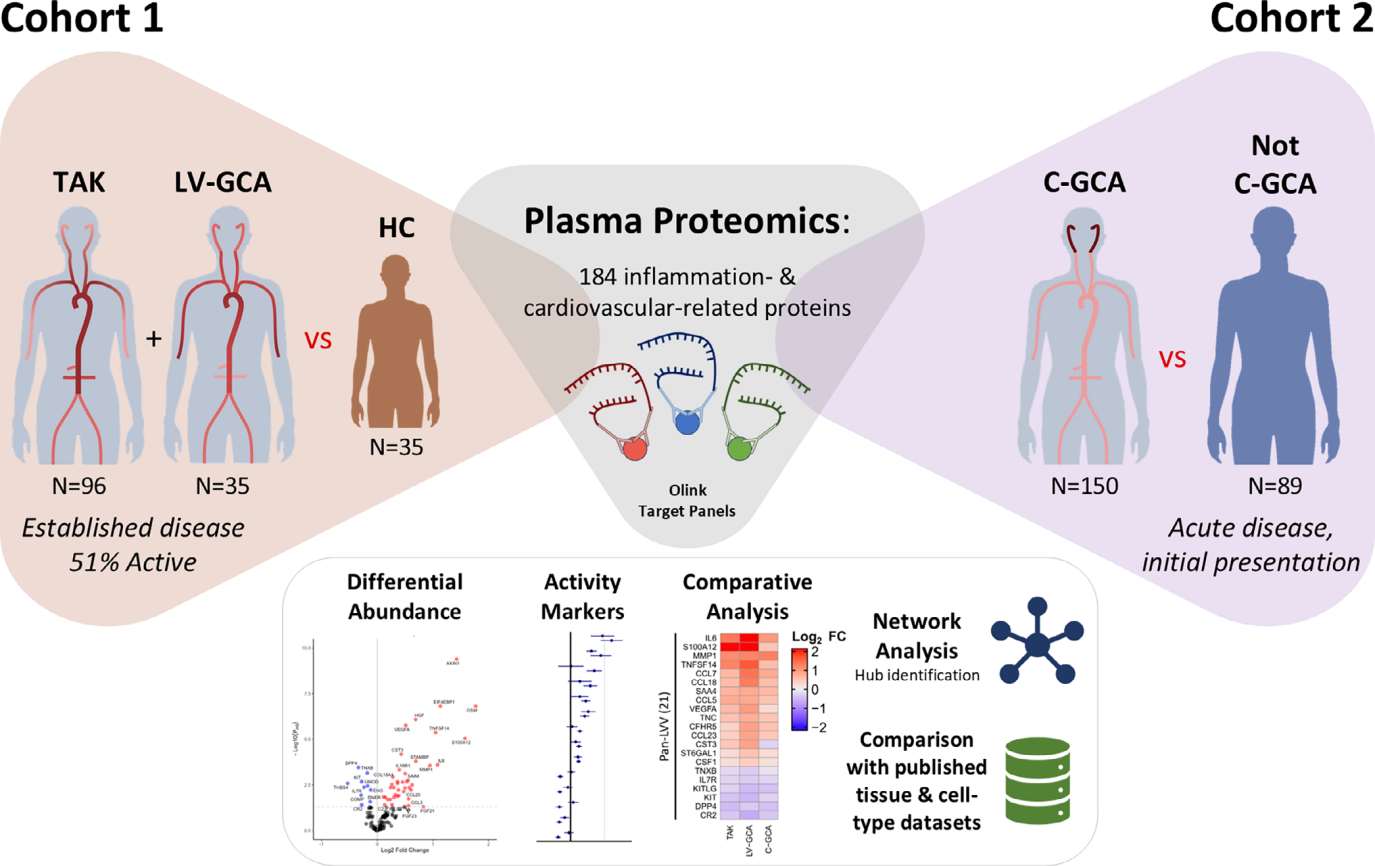
Study overview. Schematic overview of study to investigate the plasma proteomic changes associated with each form of LVV in two independent cohorts. Cohort 1: patients with established TAK and LV‐GCA and HC participants. Cohort 2: patients presenting acutely with possible cranial giant cell arteritis in whom the diagnosis was subsequently confirmed (C‐GCA) or ruled out (Not C‐GCA). Disease‐specific proteomic profiles defined by differential abundance analysis were compared. Network‐based analysis of LVV‐associated proteins and integrated analysis with published tissue and cell‐type data sets was also conducted. C‐GCA, cranial giant cell arteritis; HC, healthy control participant; ILV‐GCA, large‐vessel giant cell arteritis; LVV, large‐vessel vasculitis; TAK, Takayasu arteritis.

To identify proteins associated with LVV and to evaluate the presence of shared or distinct molecular signatures across TAK, LV‐GCA, and C‐GCA, we measured plasma levels of 184 inflammation‐ and cardiovascular‐related proteins (Supplementary Data 1) in two independent cohorts using the Olink Target antibody–based proximity extension assay (Figure 1). To provide a standardized nomenclature, we report proteins using the nonitalicized Human Genome Organisation gene symbol of the encoding gene.

### Shared plasma proteomic profiles in TAK and LV‐GCA

Cohort 1 included 96 patients with TAK, 35 patients with LV‐GCA, and 35 HCs (Supplementary Table 1). Patient characteristics were typical for TAK and LV‐GCA regarding age and sex, with younger onset and greater female to male ratio in TAK. HCs were well matched in terms of demographics to the patients with TAK. Patients with LV‐GCA were older and had a higher proportion of individuals of White European ancestry. The patient samples analyzed encompassed a broad range of disease activity, disease duration, and treatment status, particularly in TAK, in which sample size was greater. Patients with active disease tended to have shorter disease durations and were receiving higher glucocorticoid doses, as might be expected (Supplementary Data 3). In cohort 1, 158 proteins (86%) passed QC parameters (see the Methods section) and were available for analysis.

**Figure 2:**
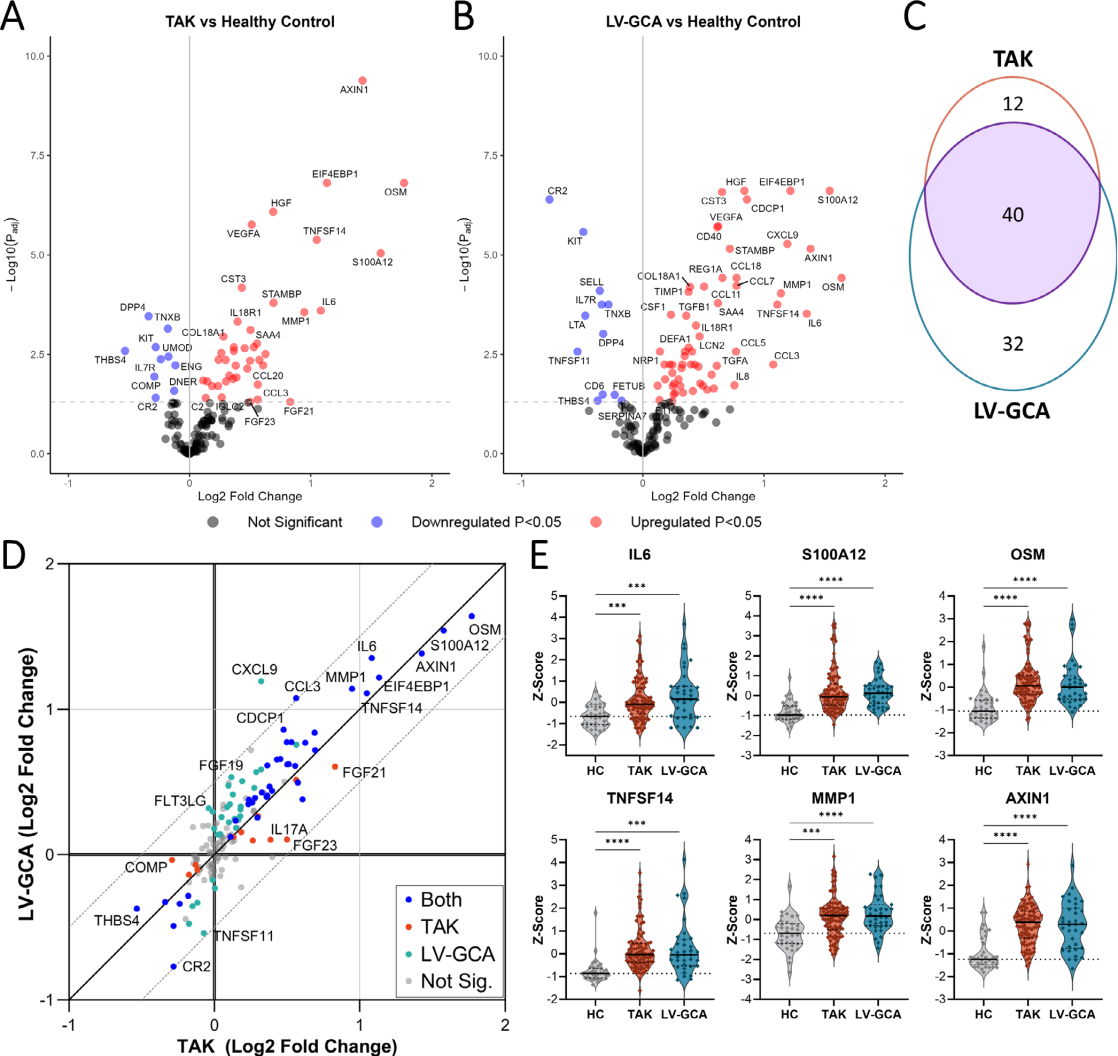
Plasma proteomic changes in TAK and LV‐GCA compared to HCs. Volcano plots showing results of differential protein abundance analyses: (A) patients with TAK (n = 96) versus HCs (n = 35), (B) patients with LV‐GCA (n = 35) versus HC. −Log10(*P*_adj_) = −log_10_ Benjamini‐Hochberg adjusted *P* value. Red and blue indicate proteins that are significantly (*P*_adj_ < 0.05) up‐regulated and down‐regulated, respectively. (C) Venn diagram showing the overlap in proteins significantly altered in TAK versus HC compared to LV‐GCA versus HC analyses. (D) Comparison of log_2_ fold changes in all proteins for TAK versus HC and LV‐GCA versus HC analyses, diagonal lines represent line of identity and ± 0.5 log_2_ fold change. Blue represents proteins that are significant in both analyses; orange represents proteins that are significant only in TAK versus HC; teal represents proteins that are significant only in LV‐GCA versus HC; gray represents proteins that are nonsignificant in both analyses. (E) Violin plots showing scaled protein levels (as Z scores) for selected proteins with prominent changes in both TAK and LV‐GCA. *P*_adj_ < 0.05: *, *P* ≤ 0.01: **, *P* ≤ 0.001: ***, *P* ≤ 0.0001: ****. HC, healthy control; LV‐GCA, large‐vessel giant cell arteritis; Sig., significant; TAK, Takayasu arteritis. Color figure can be viewed in the online issue, which is available at http://onlinelibrary.wiley.com/doi/10.1002/art.43110/abstract.

Comparison of proteomic profiles between patients with TAK and HCs identified 52 differentially abundant proteins (DAPs), with 42 up‐regulated and 10 down‐regulated (Figure 2A and Supplementary Data 4). Cross‐referencing of our results to those of a previous study,^27^ which used a different proteomic platform, demonstrated that many of our proteomic associations were novel (Supplementary Figure S1, Supplementary Data 4). We next compared LV‐GCA to HCs, revealing 72 DAPs, with 60 increased and 12 decreased (Figure 2B and Supplementary Data 5). The proteomic changes in TAK and LV‐GCA in comparison to HCs were highly similar; 40 proteins were significantly altered in both diseases, and 85% of all proteins had directionally concordant changes with respect to HCs (Figure 2C and D). Proteomic similarity of TAK and LV‐GCA was also reflected in PCA, with TAK and LV‐GCA clustering together and separated from HCs (Supplementary Figure S2A).

As expected, many up‐regulated proteins in TAK and LV‐GCA indicated immune activation including cytokines, chemokines, and growth factors (Supplementary Figure S2B). Plasma interleukin 6 (IL‐6), the pleiotropic cytokine of known importance to LVV pathogenesis,^13^ was significantly up‐regulated in both TAK and LV‐GCA (Figure 2E), together with liver‐derived inflammatory proteins such as SAA4 and FCN2. Prominent innate immune involvement in both TAK and LV‐GCA was indicated by increased levels of neutrophil‐derived proteins (S100A12, LCN2, DEFA1) and monocyte/macrophage activation and chemotactic factors (CSF1, CCL3, CCL5, CCL7, CCL14). Plasma levels of tumor necrosis factor (TNF), IL‐12B, and interferon γ were not significantly altered despite previous links to TAK pathogenesis.^28^ In contrast, we observed large increases in oncostatin M (OSM) and TNF superfamily 14 (TNFSF14) (LIGHT), cytokines not previously associated with LVV (Figure 2E). In addition to the dysregulation of immune‐related proteins, we observed the up‐regulation of proteins with functions related to the extracellular matrix (TIMP1, MMP‐1, CST3, TNC), fibrosis (TGFB1) and angiogenesis (VEGF‐A, HGF, ANG, COL18A1) in both diseases, likely reflecting a signature of arterial injury and remodeling. Six proteins were consistently down‐regulated in both diseases, including IL‐7R, KIT, TNXB, and THBS4 (both extracellular matrix [ECM]‐related glycoproteins), DPP4 (a glucose metabolism and T cell activation factor), and CR2 (the complement C3d receptor).

To evaluate whether there were disease‐specific effects, we performed a direct comparison of TAK versus LV‐GCA, which revealed six significant DAPs (Supplementary Data 6). Visualizing the relative abundance of these proteins in each group demonstrated that the dysregulation of these proteins appeared to be LV‐GCA specific (Supplementary Figure S3A). For example, in LV‐GCA, but not TAK, CXCL9, CCL11, and CA3, levels were elevated compared to HCs. Similarly, CR2 and TNFSF11 (RANKL) were significantly reduced in LV‐GCA but not in TAK. Changes specific to TAK were less prominent, with no proteins that had significant changes in TAK versus both HCs and LV‐GCA. However, we observed that FGF23 and IL‐17A were significantly increased in TAK compared to HCs but were not significantly increased in LV‐GCA versus HC (Supplementary Figure S3B).

### Signatures of active disease in patients with TAK and LV‐GCA

**Figure 3:**
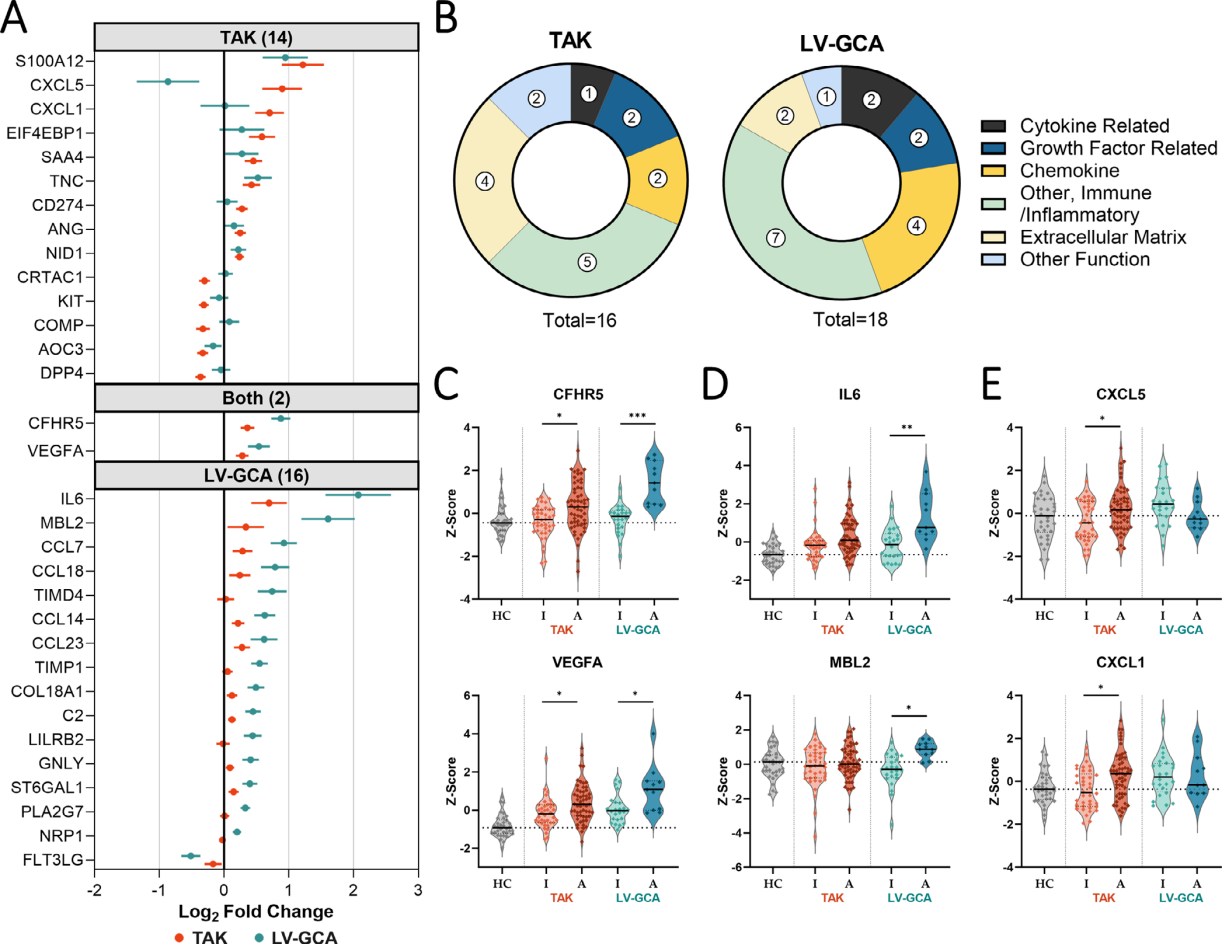
Proteins associated with active disease in TAK and LV‐GCA. (A) Points indicating log_2_ fold change estimate ± SE (horizontal bars) for proteins that were significantly (adjusted *P* < 0.05) differentially abundant in the active versus inactive patient analysis for TAK only (n = 56 vs n = 40) (top panel), both diseases (middle panel) and LV‐GCA only (n = 11 vs n = 24) (bottom panel). (B) Functional categories of differentially abundant proteins from TAK and LV‐GCA activity analyses. Violin plots depict the scaled abundance values (Z scores) for proteins associated with disease activity in (C) both diseases and (D and E) for the proteins that had divergent associations with disease activity in TAK and LV‐GCA. *P* values adjusted using Benjamini‐Hochberg method; no symbol: nonsignificant, adjusted *P < 0.05, **P ≤ 0.01, ***P ≤ 0.001. HC, healthy control; LV‐GCA, large‐vessel giant cell arteritis; TAK, Takayasu arteritis. Color figure can be viewed in the online issue, which is available at http://onlinelibrary.wiley.com/doi/10.1002/art.43110/abstract.

To identify proteins associated with disease activity in TAK and LV‐GCA, we next compared the proteomic profile of active and inactive patients within each disease. In TAK samples, 16 proteins were significantly altered in active disease, with 11 up‐regulated and 5 down‐regulated (Figure 3A and Supplementary Data 7). Up‐regulated proteins included neutrophil‐related factors (S100A12, CXCL5, CXCL1), liver‐derived proteins (SAA4, CFHR5), ECM components (TNC, NID1, CRTAC1, COMP), and angiogenic factors (VEGF‐A, ANG), indicating innate immune activation and vascular remodeling (Figure 3B). To corroborate the results of the active versus inactive patient analysis and identify proteins whose levels vary with the degree of disease activity, we tested for association with the numerical ITAS2010 disease activity score.^14^ Eight of the 158 proteins measured were significantly correlated (adjusted *P* < 0.05) with the ITAS2010 score (Supplementary Figure S4A and C), and 7 of these 8 were differentially abundant in the active versus inactive analysis.

Assessment of disease activity in TAK is currently based on the evaluation of clinical features, imaging, and clinical laboratory measures of inflammation, particularly CRP levels. However, CRP lacks both sensitivity and specificity for active TAK.^7^ In keeping with this, 20 patients with active TAK had normal CRP levels (<5 mg/L). We found that six proteins (NID1, TNC, S100A12, CD274 [soluble programmed death ligand 1], DEFA1, and DPP4) were more strongly correlated with ITAS2010 than CRP (Supplementary Figure S4). Motivated by this finding, we formally evaluated whether we could develop a multimarker protein signature using supervised learning to improve classification of disease activity (Supplementary Material). Using Least Absolute Shrinkage and Selection Operator (LASSO) regression, we generated a 10‐protein signature that outperformed CRP (area under the curve [AUC] 0.8 vs 0.72, respectively; Supplementary Figure S5A and B). However, a simpler two‐protein logistic regression model comprising CRP and COMP performed even better (AUC 0.9; Supplementary Figure S5C), demonstrating proof‐of‐principle that the addition of a single protein could provide improved and clinically tractable biomarker‐based assessment of disease activity in TAK.

In LV‐GCA, 18 proteins were significantly associated with active disease, with 17 increased and 1 decreased (Figure 3A, Supplementary Data 8). Although two proteins (CFHR5 and VEGF‐A) were increased in active disease in both LV‐GCA and TAK, the LV‐GCA activity signature was mostly distinct to that of TAK (Figure 3A and C). A more prominent acute phase response was evident in active LV‐GCA compared to TAK with large increases in IL‐6 and multiple liver‐derived inflammatory proteins (MBL2, ST6GAL1, C2) (Figure 3D). There were also differences in the chemokines and other immunoregulatory proteins affected; CXCL5 and CXCL1 levels were not significantly changed in active LV‐GCA (Figure 3E), but there were increases in the monocyte‐attracting chemokines (CCL7, CCL14, CCL23) and the T cell recruitment and activation factors (CCL18, GNLY, TIMD4). Lastly, there were activity‐associated increases in remodeling‐associated proteins TIMP1, COL18A1, and NRP1 in LV‐GCA but not TAK.

To determine whether proteomic differences persist despite clinically quiescent disease, we compared patients with inactive TAK and LV‐GCA to HCs. This analysis identified 22 and 61 DAPs in inactive TAK and LV‐GCA, respectively, with 18 DAPs being common to both diseases (Supplementary Figure S6A–C, Supplementary Data 9 and 10). Examples of proteins that remained elevated in inactive disease include OSM, S100A12, TNFSF14, and AXIN1 (Supplementary Figure S6D). Importantly, these proteins remained chronically elevated regardless of disease duration (Supplementary Figure S6E), even in patients with TAK who had withdrawn all treatment following durable clinical remission (Supplementary Figure S6D; median time off treatment 2 years [IQR 1.5–5.1 years], further details in Supplementary Data 11). Thus, our data indicate that some proteomic changes observed in patients with TAK and LV‐GCA represent persistent molecular derangements that do not normalize with clinical remission.

### Biopsy‐proven C‐GCA has a distinct proteomic endotype

We next performed proteomic profiling of an independent cohort of 239 patients presenting acutely with possible C‐GCA (cohort 2), recruited to the TABUL study.^17^ Blood samples were taken rapidly following initiation of high‐dose glucocorticoids; median treatment duration was 2 days (IQR 1–4 days). All patients underwent both TAB and USS, and a diagnosis of C‐GCA was subsequently confirmed or excluded (Not C‐GCA) as described in the Methods. Patient characteristics were typical for suspected C‐GCA, with the majority of patients being more than 60 years old (87.4%), female (72%), and having White European ancestry (99.3%) (Supplementary Table 2). Of patients diagnosed with C‐GCA, 56 patients (37.3%) had a positive TAB, 78 patients (52%) had an abnormal USS, and 53 patients (35.3%) were negative for both TAB and USS. Compared to the Not C‐GCA group, patients with C‐GCA were slightly older (median age 4 years greater) and had higher erythrocyte sedimentation rates (ESRs), CRP levels, and platelet levels (Supplementary Table 2). Proteomic profiling was performed using the same Olink platform as for cohort 1. A total of 167 proteins (91%) passed QC parameters, and data from a minimum of 225 patients were available for analysis (see the Methods section).

**Figure 4:**
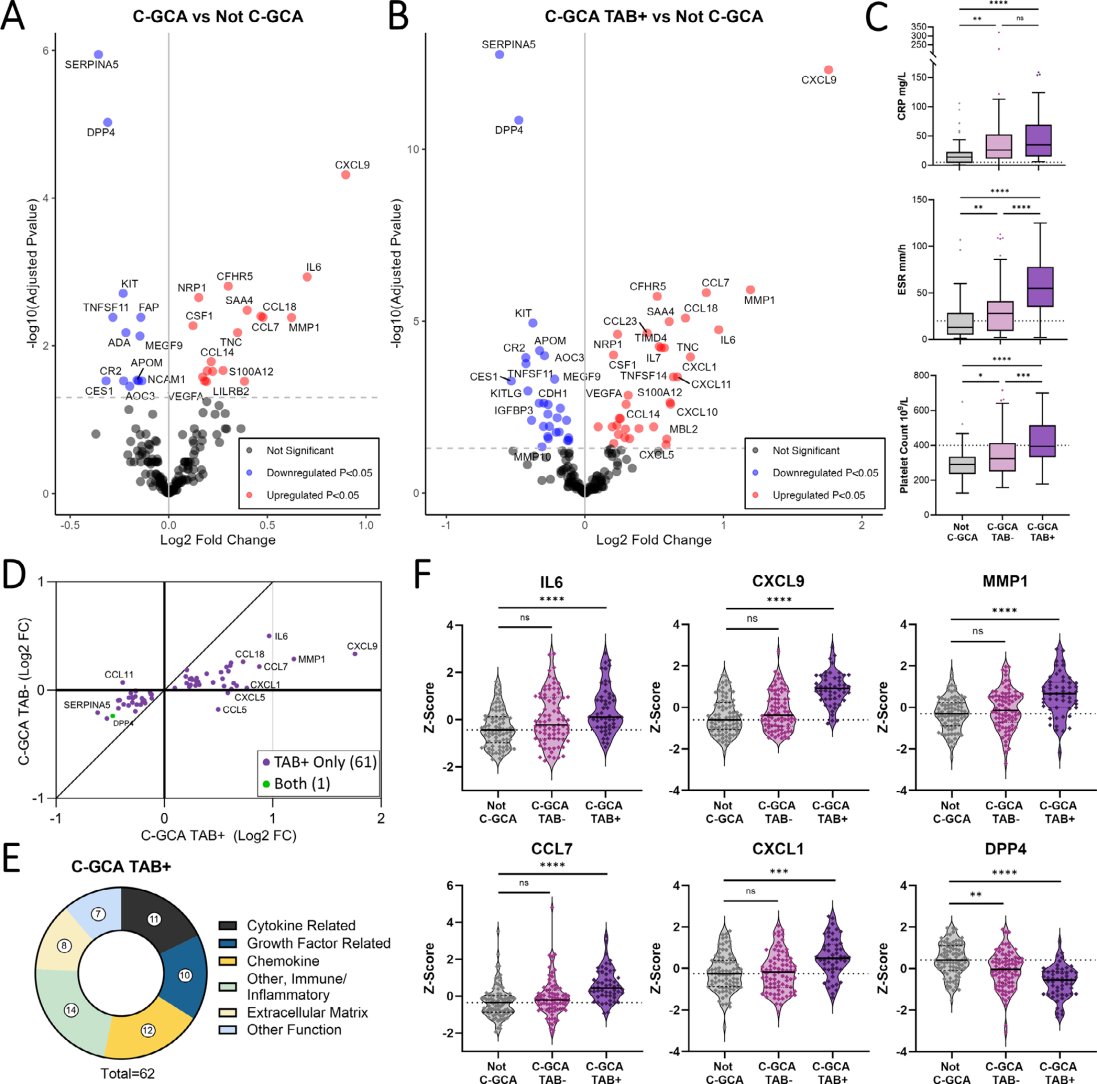
Proteomic changes associated with C‐GCA are most pronounced in biopsy‐proven disease. (A) Volcano plots for the differential protein abundance comparisons of C‐GCA (n = 150) versus Not C‐GCA (n = 89) and (B) for the comparison of TAB positive C‐GCA (C‐GCA TAB+, n = 56) versus Not C‐GCA (n = 89). −Log_10_(P_adj_) = −log_10_ Benjamini‐Hochberg adjusted *P* value. Red and blue indicate proteins that are significantly (*P*_adj_ < 0.05) up‐regulated and down‐regulated, respectively. (C) Boxplot showing CRP, ESR, and platelet count in Not C‐GCA, TAB− C‐GCA, and TAB+ C‐GCA. Median and IQR represented by line and box edges respectively, upper whisker represents the upper quartile plus 1.5 times the IQR, lower whisker represents lower quartile minus 1.5 times IQR. Statistical comparisons made with Kruskal‐Wallis and Dunn's post hoc tests. (D) Comparison of log_2_ fold changes for differentially abundant proteins in C‐GCA TAB+ versus Not C‐CGA and C‐GCA TAB− versus Not C‐GCA, diagonal line represents line of identity. (E) Functional categories of differentially abundant proteins in C‐GCA TAB+ versus not C‐GCA comparison. (F) Violin plots showing scaled protein levels (Z score) for selected proteins with prominent changes in C‐GCA TAB+ cases. Adjusted **P < 0.05, ****P ≤ 0.0001. C‐GCA, cranial giant cell arteritis; CRP, C‐reactive protein; ESR, erythrocyte sedimentation rate; IQR, interquartile range; ns, nonsignificant; TAB, temporal artery biopsy. Color figure can be viewed in the online issue, which is available at http://onlinelibrary.wiley.com/doi/10.1002/art.43110/abstract.

Proteomic comparison of C‐GCA (n = 150) to Not C‐GCA (n = 89) revealed 31 DAPs (Figure 4A and Supplementary Data 12). Further investigation revealed heterogeneity of these protein profiles within C‐GCA and that differences compared to the Not C‐GCA group were mostly driven by the TAB‐positive (TAB+) C‐GCA subgroup (Supplementary Methods, Supplementary Figure S7). In analyses stratified by TAB result, comparison of TAB+ C‐GCA (n = 56) to Not C‐GCA identified 62 DAPs (Figure 4B, Supplementary Data 13), whereas only 1 DAP was identified in the TAB‐negative (TAB−) C‐GCA (n = 89) versus Not C‐GCA comparison (Supplementary Data 14). The increase in significant associations when limiting to biopsy‐proven cases, despite reduction in sample size and statistical power, indicates that TAB+ C‐GCA is enriched for proteomic signal and that the TAB− C‐GCA group was diluting this signal in the analysis of all C‐CGA versus not C‐GCA. Comparison of the estimated log_2_FCs and protein abundances from the TAB+ and TAB− stratified analyses confirmed larger magnitudes of effect in the former (Figure 4D and F). We additionally found that patients with TAB+ C‐GCA had higher levels of CRP, ESR, platelets, and presence of polymyalgic symptoms compared to patients with TAB− C‐GCA (Figure 4C and Supplementary Data 15). Moreover, PCA of all proteins assayed indicated separation of TAB+ C‐GCA from TAB− C‐GCA (Supplementary Figure S7C). Together, these findings indicate that C‐GCA can be stratified into biologically and clinically distinct subsets by TAB result.

Further exploration using hierarchical clustering revealed that although most TAB− C‐GCA patients were proteomically distinct from patients with TAB+ C‐GCA, 18 TAB− C‐GCA patients displayed a similar profile to the TAB+ C‐GCA group (Supplementary Figure S8). However, this pattern had no significant association with demographic or clinical parameters. Relatedly, the comparison of patients with TAB− C‐GCA who had abnormal USS (n = 36) to patients without C‐GCA did not identify any significant proteins. Together, these results suggest that TAB rather than USS positivity more closely reflects the proteomic phenotype.

The 62 proteins associated with TAB+ C‐GCA suggest both innate and adaptive immune activation with dysregulation of cytokines, growth factors, chemokines, and other immune‐related proteins (Figure 4E). In a previous proteomic study,^29^ five of these proteins were identified as significantly altered in C‐GCA (Supplementary Figure S9 and Supplementary Data 13). Similar to TAK and LV‐GCA, many proteins involved in innate immune function were up‐regulated including acute phase response mediators (IL‐6, MBL2, SAA4, CFHR5, ST6GAL1) and factors involved in neutrophil and monocyte migration and activation (CXCL1, CCL14, CCL7, S100A12, CSF‐1). Several chemokines involved in T and B cell recruitment were also increased (CXCL9, CXCL10, CXCL11, CCL18) together with altered levels of lymphocyte survival and proliferation factors (increased: IL‐7; decreased: IL‐7R, KITLG, TNFSF11). In addition, increases in ECM (MMP‐1, TIMP1, TNC, LTBP2), fibrosis (TGFB1) and angiogenesis‐related proteins (VEGF‐A, HGF, NRP1) were indicative of vascular remodeling. The down‐regulated proteins with the lowest *P* values were SERPINA5 and DPP4 (Figure 4F). In secondary analyses, we did not identify any proteins that were significantly associated with cranial ischemic complications or polymyalgic symptoms within patients with C‐GCA when analyzed both as a single group and when separated by TAB result.

### Supervised learning to identify protein‐based diagnostics for C‐GCA

There are no specific diagnostic blood tests for C‐GCA. Although ESR and CRP are typically elevated, they are nonspecific. For example, CRP was elevated (>5 mg/L) in 69% of patients in the Not C‐CGA group in this study. We therefore tested whether a blood‐based protein or proteomic biomarker could aid diagnosis of C‐GCA, randomly splitting the data into training and test sets (Supplementary Material). Using logistic regression models, the best predictive univariate markers for biopsy‐proven C‐GCA were CXCL9, DPP4, and SERPINA5 (Supplementary Figure S10A, Supplementary Table 3). Despite having one of the larger estimated log_2_FCs in the differential abundance analyses (Figure 4A and B), IL‐6 was less effective as a predictor (Supplementary Table 3). We then explored whether predictive performance could be enhanced through a more sophisticated multivariate modeling approach. We supplied all proteins measured as input variables and trained a model using LASSO regression (which performs variable selection) and cross‐validation. This resulted in an 11‐protein model that provided improved diagnostic performance compared to the univariate models (accuracy 0.87, AUC 0.94) (Supplementary Figure S10B, Supplementary Tables 3 and 4). The most important features in the LASSO model were CXCL9, DPP4, and SERPINA5 (Supplementary Figure S10B), consistent with the findings of the univariate logistic regression analyses.

### Correlated proteomic changes in active TAK, LV‐GCA, and biopsy‐proven C‐GCA with IL‐6 and VEGF‐A identified as key hub proteins

We next explored similarities and differences in the plasma proteomic signatures associated with each form of LVV. We considered the possibility that our results might be impacted by differences in study design. In cohort 2, patients with C‐GCA were sampled with active disease at the time of diagnosis, whereas in cohort 1, patients were sampled during both active and inactive disease over a range of disease durations. To mitigate against this, we reanalyzed cohort 1 restricting patient samples to active disease only. These analyses revealed 68 and 69 DAPs for active TAK versus HCs and active LV‐GCA versus HCs, respectively, of which the majority had also been significantly altered in the corresponding previous analyses using all cases (80.9% and 81.6% respectively, Supplementary Data 16 and 17). We then compared the results of these TAK and LV‐GCA analyses to the proteomic associations identified in the TAB+ C‐GCA versus not C‐GCA comparison in cohort 2.

**Figure 5:**
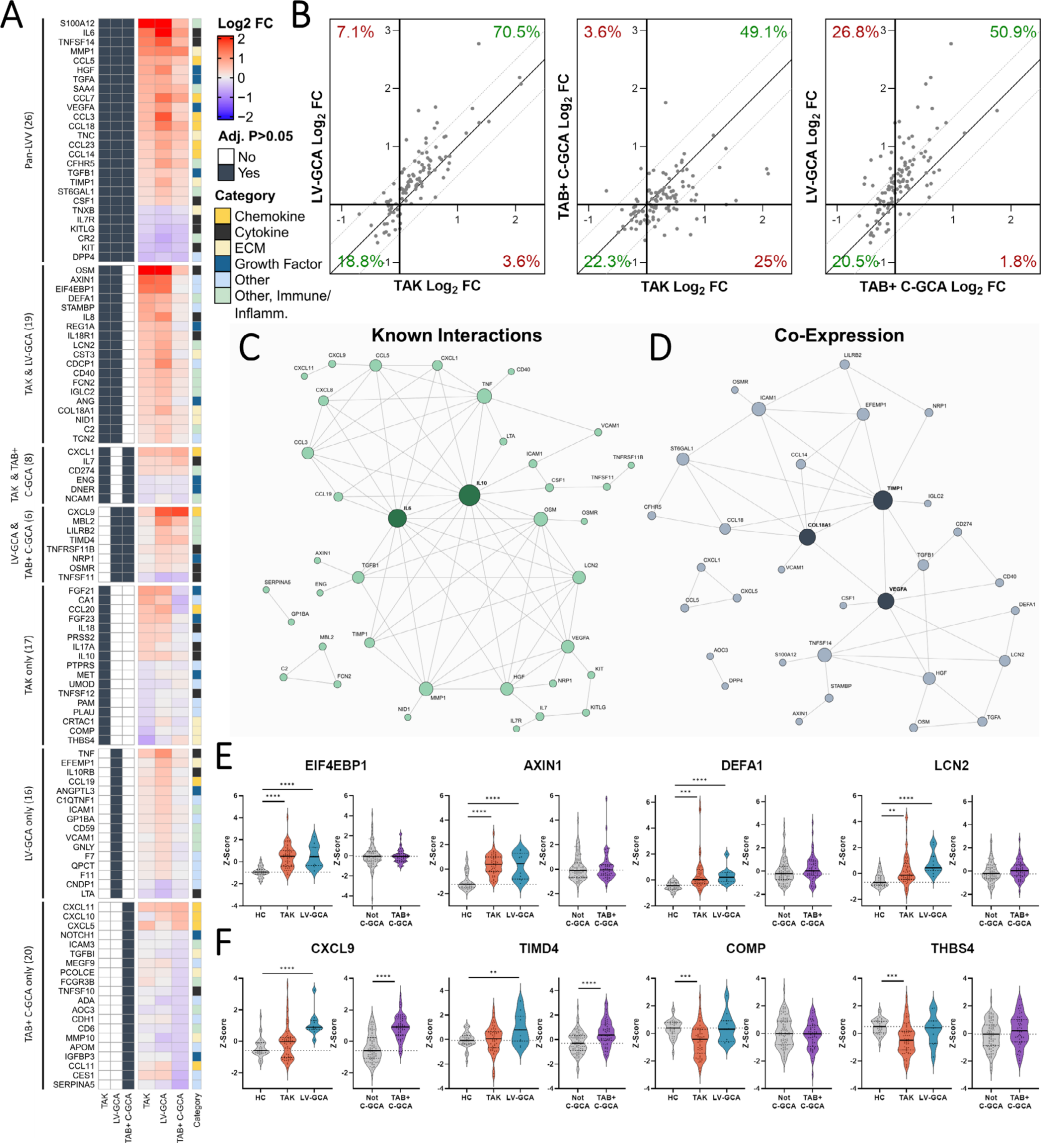
Comparison of active TAK, LV‐GCA and biopsy‐proven C‐GCA proteomic profiles. (A) Heatmap showing log_2_FCs of the 112 DAPs identified in active TAK versus HC, active LV‐GCA vs HC, and TAB+ C‐GCA versus not C‐GCA comparisons. Left: navy boxes represent proteins with statistically significant changes (adjusted *P* < 0.05) in each disease. Right: annotated functional category for each protein. (B) Comparison of disease vs HC log_2_FCs for 112 DAPs. Diagonal lines represent the line of identity. Network plots of (C) known protein–protein interactions and (D) protein–protein coexpression for 74 proteins with directionally concordant changes across diseases. Node size corresponds to number of edges. For C, high confidence interactions (≥0.9) were sourced from STRING^19^ and for D, protein coexpression was defined as a Pearson correlation ≥0.6 in both patients with active TAK/LV‐GCA and patients with TAB+ C‐GCA. (E) Violin plots showing scaled abundance values (Z scores) for selected proteins associated with active TAK and LV‐GCA but not C‐GCA and (F) those identified as different between active LV‐GCA and C‐GCA versus active TAK. *P* values adjusted using Benjamini‐Hochberg method; no symbol: nonsignificant, Adjusted *P < 0.05, **P ≤ 0.01, ***P ≤ 0.001, ****P ≤ 0.0001. C‐GCA, cranial giant cell arteritis; CRP, C‐reactive protein; DAP, differentially abundant protein; ESR, erythrocyte sedimentation rate; HC, healthy control participant; IQR, interquartile range; log_2_FC, log 2 fold change; LV‐GCA, large‐vessel giant cell arteritis; ns, nonsignificant; TAB, temporal artery biopsy; TAK, Takayasu arteritis. Color figure can be viewed in the online issue, which is available at http://onlinelibrary.wiley.com/doi/10.1002/art.43110/abstract.

A total of 112 DAPs were identified in one or more LVV type (subsequently referred to as LVV‐associated proteins, Figure 5A, Supplementary Data 18). Directional changes were highly similar with 74 (66.1%) having concordant changes (Figure 5B) and significant correlation between both TAK and LV‐GCA profiles with that of TAB+ C‐GCA (Pearson r = 0.49 and r = 0.69, respectively, both *P* < 0.0001). Twenty‐six proteins (23.2%) were dysregulated in all three diseases and 33 proteins (29.5%) were dysregulated in two. Of the 26 shared DAPs, all had directionally concordant changes, including 20 up‐regulated and 6 down‐regulated proteins. We define these 26 proteins as the “pan‐LVV signature” (Supplementary Data 18). Up‐regulated proteins included IL‐6, acute phase proteins (SAA4, CFHR5, ST6GAL1), monocyte and neutrophil factors (S100A12, CSF‐1), monocyte and lymphocyte chemokines (CCL5, CCL7, CCL3, CCL18, CCL23), and TNFSF14. Levels of proteins related to arterial remodeling were also increased, including VEGF‐A, HGF, MMP‐1, TIMP1, and the ECM glycoprotein TNC. Down‐regulated proteins included IL‐7R, KIT, and KITLG, each involved in lymphocyte differentiation and proliferation, DPP‐4, CR2, and TNXB (another ECM tenascin).

Using the GTEx tissue transcriptome database,^21^ we explored the global tissue expression profile of each LVV‐associated protein, defining enhanced expression as more than four‐fold higher than average tissue level as per Human Protein Atlas methodology.^22^ A total of 83 proteins (74.1%) had enhanced expression in ≥1 tissue. The most represented tissues were liver, spleen, and whole blood (Supplementary Figure S11). A total of 11 proteins had enhanced arterial expression including TNC, TIMP1, COL18A1, and TNFRSF11B (osteoprotegerin), thereby indicating the possibility of blood‐based measurement of arterial biomarkers in LVV.

To infer relationships between proteins, we constructed two networks using the 74 proteins with concordant changes using (1) annotated protein–protein interactions from STRING^19^ (Figure 5C) and (2) coexpression (Pearson r ≥ 0.6) (Figure 5D). The network of annotated interactions identified IL‐6 and IL‐10 as the central hubs with connections to proteins with distinct functions including chemotaxis (eg, CCL5, CCL3), angiogenesis (eg, VEGF‐A, HGF) and tissue remodeling (eg, MMP‐1, TIMP1, TGFB1). In the network constructed using protein coexpression data, TIMP1 and VEGF‐A displayed even greater connectivity, appearing as central nodes with edges connecting both to other remodeling‐related proteins (eg, COL18A1, NRP1) and multiple immune‐associated proteins (eg, CSF1, TNFSF14, CCL14). These networks indicate the coordinated regulation of immune and vascular remodeling processes, highlighting immune‐stromal cross‐talk in LVV.

The most prominent inter‐disease differences diseases were between TAK and LV‐GCA profiles compared to TAB+ C‐GCA (Figure 5A). In particular, the large increases in EIF4EBP1 and AXIN1 observed in TAK and LV‐GCA were not seen in C‐GCA. Similarly, increases in the neutrophil proteins DEFA1 and LCN2 were observed exclusively in TAK and LV‐GCA (Figure 5E). There were also differences between LV‐GCA and TAB+ C‐GCA profiles compared to TAK, which indicate some degree of divergence between diseases. For example, increases in CXCL9 and TIMD4 were seen only in the GCA groups, whereas decreases in the ECM‐related proteins COMP and THBS4 were only found in TAK (Figure 5F).

### Plasma proteomic signatures reflect LVV arterial tissue phenotype

**Figure 6:**
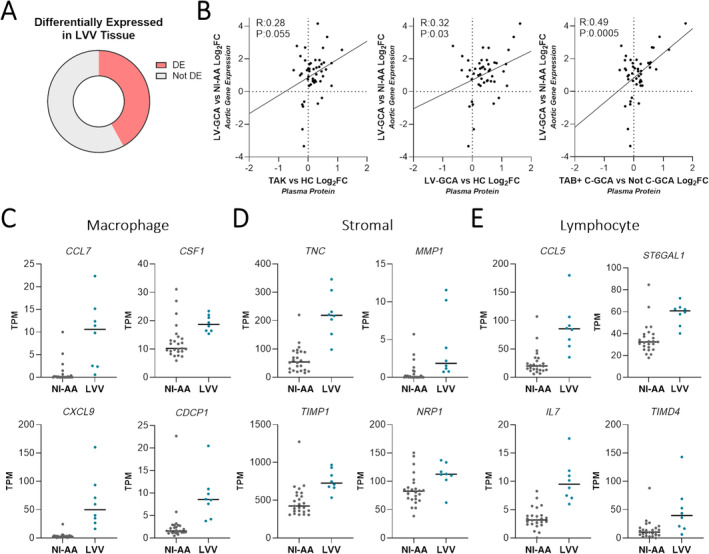
Correspondence of LVV plasma profile to arterial tissue phenotype. Comparison of plasma proteomic profiles associated with LVV and gene expression changes identified in aortic tissue affected by LVV.^23^ (A) Pie chart showing the proportion of 112 LVV‐associated plasma proteins (Supplementary Data 18) that were also differentially expressed (adjusted *P* < 0.05) in LV‐GCA‐related aortic aneurysm (n = 8) versus NI‐AA (n=25) by bulk RNA sequencing. (B) Comparison of plasma protein and aortic gene expression log_2_FCs for the 47 proteins and corresponding genes that had significant changes in both LVV plasma and aortic tissue, each point represents a gene/protein pair. For proteomics (Y axes), log_2_FCs represent active TAK versus HC, active LV‐GCA versus HC, and TAB positive C‐GCA (TAB+ C‐GCA) versus Not C‐GCA comparisons. For transcriptomics (X axes), log_2_FCs represent LV‐GCA associated aortitis versus NI‐AA. (C–E) Dot plots showing aortic gene expression of genes/proteins differentially expressed/abundant in both LVV plasma and aortic tissue. Aortic gene expression in LVV versus NI‐AA for genes typically expressed by (C) macrophages, (D) nonhematopoietic stromal cells, and (E) lymphocytes. Gene expression measured in TPM, cell‐type expression classified using blueprint data (Figure S8). *P* values were adjusted using Benjamini‐Hochberg method. C‐GCA, cranial giant cell arteritis; HC, healthy control participant; log_2_FC, log_2_ fold change; LV‐GCA, large‐vessel giant cell arteritis; LVV, large‐vessel vasculitis; NI‐AA, noninflammatory aortic aneurysm; TAB, temporal artery biopsy; TAK, Takayasu arteritis; TPM, transcripts per million. Color figure can be viewed in the online issue, which is available at http://onlinelibrary.wiley.com/doi/10.1002/art.43110/abstract.

Plasma proteins arise not only from blood cells but also from a wide range of tissues and organs, including the vasculature, which is in direct contact with the blood. We therefore sought to evaluate whether changes in patient plasma (Figure 5A) reflect the phenotype of arterial tissue affected by LVV. Using bulk RNA‐seq data from a study that compared surgically resected aortic tissue of LV‐GCA to noninflammatory aortic aneurysms (NI‐AA),^23^ we found that for 47 (42%) of the LVV‐associated plasma proteins, the corresponding gene was differentially expressed in LVV arterial tissue (Figure 6A and Supplementary Data 19). Moreover, the log_2_FCs of these 47 plasma proteins correlated with the log_2_FC of the corresponding gene in LVV tissue versus NI‐AA (Figure 6B), and 28 plasma proteins (59.6%) had directionally concordant changes across the transcriptomic analysis of LVV arterial tissue and the plasma proteomic analyses of all the three LVV types.

Using the blueprint and GTEx bulk RNA‐seq data sets,^21^, ^30^ we explored the cell type and arterial expression profile of the 47 genes and proteins dysregulated in both plasma and arterial tissue (Supplementary Figure S12A). Macrophage and neutrophil expressed genes and proteins made up the largest subset (34%); this included *CCL7*, *CSF1*, *CXCL9*, and *CDCP1*, which were increased in both tissue and plasma (Figure 6C). Genes and proteins expressed by nonimmune stromal cells, such as *TNC*, *MMP1*, *TIMP1*, and *NRP1*, were also prominent (Figure 6D). This latter cluster was enriched for genes of high arterial expression (GTEx) and could be useful as markers of arterial remodeling. The remainder were lymphocyte‐derived (Figure 6E) or had mixed expression profiles. Of note, despite the significant decreases in plasma protein DPP4, CR2, and IL‐7R in LVV, the expression of the corresponding genes was significantly increased in LVV arterial tissue (Figure S12B), emphasizing that there may be directionally discordant effects between different tissue compartments and/or between intracellular messenger RNA (mRNA) expression and plasma protein levels. Overall, these findings indicate that plasma proteomic signatures can reflect aspects of LVV tissue phenotype and could provide a valuable noninvasive read‐out of pathogenic processes occurring in diseased arteries.

## DISCUSSION

TAK and GCA are currently classified as separate diseases,^12^, ^31^ but some investigators have proposed that they could represent varying manifestations of the same disease spectrum.^4^, ^5^ This debate has largely focused on phenotypic similarities, reflecting patterns of arterial injury.^3^ Genome‐wide association studies reveal differences in the genetic risk factors associated with TAK or GCA.^32^ However, a key unanswered question is whether the molecular effector pathways acting in these diseases are shared or distinct. This information is important for the rational selection of new therapeutic strategies that target specific proteins.

Here, we address this by comparing the plasma proteomic profile of TAK, LV‐GCA, and C‐GCA. In 281 patients with LVV, we measured 184 inflammation‐ and vascular‐associated proteins to characterize the plasma proteome of each major LVV type and evaluate changes associated with disease activity states. We found that the proteomic profiles associated with active TAK, LV‐GCA, and biopsy‐proven C‐GCA were similar and identified a 26‐protein “pan‐LVV” signature common to all three groups. This signature primarily included proteins of immunologic function, but it also comprised proteins arising from or acting on the stroma, indicative of arterial injury, and/or repair. Some of these proteins have well‐established roles in LVV (eg, IL‐6 and VEGF‐A),^13^, ^28^ but others have not been previously linked to LVV.

The signature reflected prominent innate immune activation, particularly with increases in several proteins related to monocyte and macrophage function. Coexpression analysis revealed coordinated regulation of such proteins (eg, CSF1, CCL18, CCL14) with multiple proteins involved in tissue remodeling (eg, VEGF‐A, TIMP1, and TGFB1) thereby highlighting innate immune‐stromal cross‐talk in LVV. This is consistent with recent reports implicating profibrotic macrophage subsets in the fibrotic and stenotic remodeling of arteries in TAK and GCA, cell types that are less affected by current treatments.^33^, ^34^ These findings underscore the importance of macrophages in TAK and GCA pathogenesis and illustrate the need for therapeutics which target macrophages beyond proinflammatory functions alone.

We also observed evidence for adaptive immune involvement, with changes in lymphocyte‐related proteins such as IL‐7R and TNFSF14. Plasma IL‐7R was decreased in all LVV groups irrespective of disease activity status. Its ligand IL‐7 was also significantly increased in TAK and C‐GCA. IL‐7 is essential for T cell development and homeostasis while soluble IL‐7R (sIL‐7R) potentiates IL‐7 activity by enhancing bioavailability.^35^ The pattern observed is similar to findings in antineutrophil cytoplasmic antibody (ANCA)–associated vasculitis and tuberculosis infection but contrasts with rheumatoid arthritis and lupus in which sIL‐7R levels are increased.^36^, ^37^, ^38^ Interestingly, the mRNA expression of IL‐7R was increased in the transcriptomic analysis of LV‐GCA aortitis, and a recent study reported that IL‐7R expressing T cells are involved in the persistence of vasculitic lesions in a mouse chimeric model.^39^ Decreased IL‐7R in the plasma of patients with LVV may therefore reflect the recruitment of specific T cell subsets from the circulation to vasculitic lesions. As another example, TNFSF14 (LIGHT) was prominently elevated. TNFSF14 acts as a T cell costimulatory factor and triggers T cell activation and proliferation.^40^ TNFSF14 promotes systemic immunopathology, as demonstrated by transgenic animals with constitutive TNFSF14 expression in T cells.^41^ Moreover, TNFSF14 can also act on nonhematopoietic structural cells including fibroblasts, endothelial, and smooth muscle cells to drive tissue fibrosis.^42^ Consistent with this, our coexpression network analysis identified an edge connecting TNFSF14 to VEGF‐A, a central hub node. Current treatment strategies in LVV are limited to suppression of inflammation and do not target fibrosis directly. Thus, TNSF14 antagonism may be a novel therapeutic approach in LVV that provides dual targeting of both the immune response and the consequent stromal reaction.

Lastly, the signature included tissue remodeling proteins expressed by immune cells (eg, VEGF‐A) or stromal cells (eg, TNC, TIMP1, MMP‐1); the latter may represent useful markers of arterial damage independent of inflammation. Increased plasma TNC in LVV is relevant given its enrichment in normal arteries and increased expression in arteries affected by LVV. TNC is an ECM protein primarily expressed by fibroblasts at sites of tissue damage where it supports repair. It gained interest as a candidate marker of vascular injury, but its levels are also elevated in other disease states.^43^ Although this lack of specificity to LVV may preclude its use as a diagnostic marker, TNC, and other ECM proteins could be useful for monitoring arterial injury during follow‐up and should be evaluated in longitudinal studies.

Despite the overall similarity in LVV proteomic profiles, we identified differences that suggest some immunologic divergence between diseases. For example, the chemokine CXCL9 was higher in LV‐GCA and C‐GCA compared to TAK. CXCL9 is produced by macrophages and other cell types in response to interferon γ and typically reflects activation of the Th1 pathway.^44^ Increased plasma CXCL9 has been reported previously in GCA and is associated with CXCR3+ cell infiltration into diseased arteries.^45^ Previous studies speculated that TAK and GCA differ in the susceptibility of T cell pathways to glucocorticoids whereby Th1 responses persist in GCA and Th17 responses persist in TAK after treatment initiation.^46^, ^47^ Our observation that CXCL9 was exclusively increased in GCA while IL‐17A was exclusively increased in TAK may support this theory.

Beyond comparing LVV types, this study provided several disease‐specific insights. We identified novel markers of active disease in TAK and in LV‐GCA. Disease activity assessment is challenging in LVV, in which existing markers such as CRP have well‐recognized limitations, and there can be practical constraints regarding the frequent use of vascular imaging.^7^, ^8^ In a proof‐of‐principle analysis, we demonstrate that a single additional marker, the ECM glycoprotein COMP, used in combination with CRP markedly increases the accuracy of disease activity detection when compared to the use of CRP alone. Although further study is required to validate the novel markers identified here in independent cohorts, our findings suggest that significant improvements can be made with simple additions to the laboratory testing regimens used for monitoring LVV. Importantly, we also found that many proteins remained altered in patients with TAK and LV‐GCA despite clinically quiescent disease. OSM and AXIN1 were elevated regardless of disease activity, whereas activity markers such as S100A12 and VEGF‐A were highest in active disease but remained increased despite clinical disease quiescence. These findings may parallel the inaccuracy of clinical disease activity assessment compared to histopathologic or radiologic evaluations of arterial inflammation.^48^, ^49^, ^50^ However, we observed similar derangements in patients with TAK who had achieved durable clinical remission (defined here as the absence of any symptom, sign, laboratory feature or radiographic evidence for active TAK over the last three years of monitoring and the safe cessation of all immunosuppressive treatments). Therefore, although the persistent changes observed likely indicate subclinical inflammation, future studies are required to determine whether they are associated with clinically important outcomes.

Our results suggest that biopsy‐positive and ‐negative C‐GCA are proteomically distinct. There are two possibilities that could explain our findings. First, despite a careful clinical phenotyping algorithm, which included external expert review, there may be instances of misclassification within the TAB− C‐GCA group, such that some patients were erroneously labeled as C‐GCA. However, this cannot fully explain our findings because the TAB− C‐GCA group included some patients with a positive USS. Alternatively, TAB positivity may reflect the burden of arterial disease. Skip lesions, with discontinuous segments of arterial inflammation, can occur in GCA, and so a patient with lower burden of arterial disease is more likely to have a negative biopsy on the small biopsy section of artery sampled. Thus, it is possible that the proteomic read‐out may reflect quantitative differences in the extent of arteritis. Our clinical laboratory data provided additional evidence of differences between TAB+ and TAB− patients, with higher ESR, CRP, and platelet count in the former group, in keeping with previous studies.^51^, ^52^, ^53^ Other clinical differences in TAB+ and TAB− C‐GCA have been described, including greater risk of visual loss^53^, ^54^ and higher prevalence of PMR in TAB+ patients,^51^ although these findings have not replicated in all case series.^55^ Our data support the concept that C‐GCA can be stratified by biopsy into biologically distinct endotypes, which may have implications for future trial design and precision medicine strategies.

Our study had limitations. We used a targeted proteomic panel that was enriched for inflammatory and vascular proteins and thus our ability to compare between LVV groups is limited to the proteins measured. The HCs in cohort 1 were well matched to patients with TAK but were younger than patients with LV‐GCA. Differences in study design between cohort 1 and cohort 2 mean that proteomic differences between C‐GCA and the other LVV groups may be confounded by differences in disease duration and treatment history. In addition, the controls in cohort 2 (“Not GCA”) were individuals who presented with symptoms for which a diagnosis of C‐GCA was considered but ultimately excluded, whereas the controls in cohort 1 were healthy participants with no symptoms. In cohort 1, there was heterogeneity in treatment, with varying administration of steroids and other immunosuppressants, which could potentially impact the plasma proteome. However, treatment (particularly glucocorticoid dose) is given in response to disease activity, reflected in the correlation between disease activity scores and prednisolone dose in cohort 1. Thus, attempting to statistically adjust for treatment risks overadjustment.^56^, ^57^ For supervised learning, we split the data into training and test sets, but the relatively modest sample sizes mean that estimates of model performance are more vulnerable to stochastic variation. We mitigated this by also estimating model performance through cross‐validation in the larger training set. Finally, in the case of membrane‐bound proteins that undergo cleavage to produce a soluble form, it is not always clear whether plasma protein measurements are exclusively capturing the latter or also protein from cell membranes (for example, arising from in vivo sources such as exo‐ or ectosomes or ex vivo processes such as venepuncture or sample processing), complicating interpretation.

In conclusion, similarities in the plasma proteomic profiles of active TAK and GCA indicate common effector pathways resulting in inflammatory arterial damage despite differences in genetic etiology. Our integrated analysis of plasma and arterial tissue highlight the role played by macrophages and their protein products in LVV and indicate significant potential for their targeting in novel treatments and biomarkers. Future work should expand the molecular characterization of the LVV disease spectrum by extending the number of proteins measured via use of complementary proteomic platforms and by concurrent measurement of other ‐omic layers (eg, RNA‐seq of immune cells). Longitudinal studies characterizing the temporal changes in the molecular profile across the disease course will be valuable in delineating acute from chronic changes and allowing intraindividual assessment of putative biomarkers identified here.

## AUTHOR CONTRIBUTIONS

All authors contributed to at least one of the following manuscript preparation roles: conceptualization AND/OR methodology, software, investigation, formal analysis, data curation, visualization, and validation AND drafting or reviewing/editing the final draft. As corresponding author, Drs Maughan, Morgan, and Peters confirm that all authors have provided the final approval of the version to be published and take responsibility for the affirmations regarding article submission (eg, not under consideration by another journal), the integrity of the data presented, and the statements regarding compliance with institutional review board/Declaration of Helsinki requirements.

## Supporting information


Disclosure form

**Appendix S1:** Supplementary Information

Data S1:
