# Supplementary material for: Proteomic Profiling of the Large‐Vessel Vasculitis Spectrum Identifying Shared Signatures of Innate Immune Activation and Stromal Remodeling
Source: Arthritis Rheumatol. 2025 Feb 27;77(7):884–900. doi: 10.1002/art.43110 (PMC12209745; doi:10.1002/art.43110)
Supplement: Supplementary file 2 — Appendix S1: Supplementary Information [file ART-77-884-s001.docx]

**Supplementary Material**

‘Proteomic profiling of the large vessel vasculitis spectrum identifies shared signatures of innate immune activation and stromal remodelling’ by Maughan *et al*.

**Table of Contents**

1) Supplementary Methods

2) Supplementary Tables

3) Supplementary Figures

# **Supplementary Methods**

## Comparison with previous proteomic studies

The results from the case versus control analyses for Takayasu arteritis (TAK) and cranial giant cell arteritis (C-GCA) were compared to the results of the largest comparable published plasma proteomic studies with regards sample size and number of proteins measured. For TAK, this was the study of Cui et al which identified 18 TAK associated proteins using a 440-protein array^1^. In this array there were 87 proteins which overlapped with our Olink panels, and of these, 3 (CCL3, TIMP1, COMP) were identified as differentially abundant in both studies with concordant directions of effect (**Figure S1** & **Supplementary Data 4**). For C-GCA, it was the study of Burja et al which measured 49 proteins using a combination of ELISA and Luminex immunoassays^2^, of which 27 were significantly associated with C-GCA. There were 21 proteins that overlapped with our Olink panels, and of these 5 were identified as significantly upregulated compared to controls in both studies (C-GCA TAB+ vs Not C-GCA comparison, **Figure S9** & **Supplementary Data 13**).

## Association with disease activity, extended Takayasu Arteritis analysis

The association of each protein with Indian Takayasu Clinical Activity Score (ITAS2010) in TAK patients was tested using spearman correlation. For comparison, the correlation of CRP and pentraxin-3 (PTX3) with ITAS2010 was also tested since both are established markers of TAK disease activity^3^. CRP was measured in the local hospital’s clinical laboratory while PTX3 was measured using a commercially available ELISA (R&D Systems DY1826). Spearman correlation was used given the non-normal distribution of ITAS2010 scores and the resultant P values were adjusted for multiple testing using the Benjamini-Hochberg method (Adjusted P < 0.05).

## Exploring heterogeneity within C-GCA

The proteomic comparison of C-GCA patients with Not C-GCA cases identified 31 differentially abundant proteins (DAP). To identify patient subsets with the greatest changes, the Euclidean distance for each C-GCA patient to the mean of Not C-GCA cases for the 31 DAPs was calculated. Multiple linear regression analysis was then conducted to identify clinical parameters independently associated with the Euclidean distance or degree of change in the 31 DAPs. The following clinical parameters at presentation were tested for association: temporal artery biopsy (TAB) result, temporal artery ultrasound sonography (USS) result, cranial ischaemic complications, age, sex and polymyalgic symptoms. The apparent association of TAB result with proteomic profile was further confirmed within C-GCA patients using principle component analysis (PCA) of scaled abundance of proteins that passed quality control checks (N=167). Due to methodological constraints, only C-GCA patients with complete proteomic and clinical parameter data were included in regression and PCA analyses (N=133).

62 DAPs were identified in the comparison of C-GCA TAB+ patients to Not C-GCA cases while only a single protein was significantly altered in the comparison of C-GCA TAB- patients to Not C-GCA cases. To investigate the potential presence of C-GCA TAB- patients with proteomic profiles similar to TAB+ patients, patient level changes in protein abundance for the 62 TAB+ DAPs compared to the Not C-GCA mean were calculated. Then, hierarchical clustering was conducted using Euclidean distance and complete linkage using the ComplexHeatmap package in R. The resulting cluster pattern and association with clinical parameters was visually evaluated using the heatmap plot (**Figure S6**). Again, only C-GCA patients with complete proteomic and clinical parameter data were included in clustering analysis (N=133).

## Evaluating biomarker performance for disease activity assessment in TAK

Within TAK patients, 16 proteins were significantly associated with disease activity status (**Figure 3A**, **Supplementary Data 7**). The performance of each of these proteins as a biomarker of disease activity was assessed and compared to that of CRP (measured in the clinical laboratory) individually and in combination with a series of supervised learning models. TAK patients with complete proteomic data (N=94) were randomly split into Training and Test sets with an approximate 75%/25% split, ensuring a similar ratio of active to inactive cases in each set (using the *createDataPartition* function from the R caret package). This resulted in the Training set consisting of N=42 active and N=29 inactive patients, and the Test N=14 active and N=9 inactive patients. Model fitting was performed in the Training set using three alternative approaches:

1. Univariate logistic regression of each of the 17 markers vs disease activity status (**Figure S5A**)
2. Least Absolute Shrinkage and Selection Operator (LASSO) multivariate regression of all 17 markers vs disease activity status (**Figure S5B**)
3. Two-variable logistic regression of CRP plus one additional marker vs disease activity status (i.e. to assess the impact of adding each marker individually to CRP) (**Figure S5C**).

Analyses were performed in R using a combination of the caret and glmnet packages^4,5^. All models were trained using 5-fold cross-validation to avoid overfitting and to allow estimation of performance on the Training data. We performed receiver operating characteristic (ROC) curves and area under the curve (AUC) analyses. Final model performance was judged using the Test set, which had not been seen by the models during training. We computed confusion matrices comparing model predictions to the true class labels in the Test set, and from this derived performance metrics.

**Evaluating biomarker performance for C-GCA diagnosis**

We used supervised learning to develop protein-based biomarkers for the diagnosis of C-GCA. The design of Cohort 2 was optimal for evaluating this, since all patients had presented with a clinical suspicion of C-GCA which was subsequently either confirmed or excluded. Thus, we were able to evaluate discriminative performance in a relevant clinical context. To avoid any diagnostic ambiguity, we elected to use only biopsy-proven (‘TAB+’) C-GCA as ‘gold standard’ C-GCA cases and developed protein-based predictive models that discriminate these patients from Not C-GCA. Since the supervised learning techniques employed require complete data, we excluded individuals with missing protein measurements. This resulted in a dataset of 168 proteins for 135 individuals (82 Not C-GCA and 53 TAB+ C-GCA).

We then randomly split the data into Training and Test sets, ensuring a similar ratio of C-GCA to Not C-GCA in each set using the R caret package’s *createDataPartition* function (Training set: N=58 Not C-GCA and 38 TAB+ C-GCA, Test set: N=24 Not C-GCA, N=15 TAB+ C-GCA).

Using the Training set, we fit univariate (single protein) logistic regression models for each protein with 5-fold cross-validation (R caret package). Cross-validation enabled estimation of model performance on the Training set. Six proteins (SERPINA5, AOC3, CXCL9, MMP1, DPP4 and KIT) achieved accuracy >0.6 and Cohen’s kappa >0.4, and were taken forward for evaluation in the Test set. We used the *caret* and the *pROC* R packages to calculate performance metrics and to perform ROC-AUC analyses. In addition, we evaluated the performance of IL6 given its known importance in the pathogenesis of GCA.

We next used LASSO to develop a multi-protein diagnostic signature for C-GCA using all proteins measured as the input data and 5-fold cross-validation in the Training set as described in the previous analyses. Again, final model performance was judged using the Test set as described above in the TAK activity biomarker analysis.

## Exploring the expression of dysregulated plasma proteins in immune and stromal cell types

To investigate the expression of plasma proteins in human immune and stromal cell types, bulk RNA-seq data was accessed from the Blueprint consortium as fragments per kilobase per million mapped reads (FPKM) values^6^. Accessed cell types included: blood outgrowth endothelial cells, mesenchymal stem cells, monocytes, macrophage, neutrophils, basophils, eosinophils, natural killer cells, B-cells, CD4+ T-cells, CD8+ T-cells and T-regs. The expression of each gene/protein per cell-type was summarised as median and explored using hierarchical clustering analysis as described in previous sections.

# **Supplementary Tables**

## Supplementary Table 1: TAK and LV-GCA cohorts

| ​ | **HC**​ | **TA​K** | **LV-GCA**​ |
| --- | --- | --- | --- |
| N​ | 35​ | 96​ | 35​ |
| Female (%)​ | 31 (88.6)​ | 91 (94.8)​ | 28 (80)​ |
| Age​ (years) | 38.2 [30.4, 52.5]​ | 41.6 [30.9, 55.6]​ | 67.2 [61.3, 72.3]​ |
| Self-identified Ethnicity​ | ​ | ​ | ​ |
| White European​ (%)​ | 21 (60)​ | 55 (57.3)​ | 30 (85.7)​ |
| Asian​ (%)​ | 11 (31.4)​ | 35 (36.5)​ | 4 (11.4)​ |
| Other​ (%)​ | 3 (8.6)​ | 6 (6.3)​ | 1 (2.9)​ |
| Time since diagnosis, yrs​ | -​ | 3.9 [1.1, 10.3]​ | 1.4 [0.5, 3.7]​ |
| Active Disease​ (%)​* | -​ | 56 (58.3)​ | 11 (31.4)​ |
| CRP >5mg/L​ (%)​ | -​ | 42 (43.7)​ | 17 (48.6)​ |
| ESR >20mm/hr (%)​​ | -​ | 59 (61.5)​ | 17 (48.6)​ |
| Treatment​ | ​ | ​ | ​ |
| No treatment (%)​​ | -​ | 21 (21.9)​ | 6 (17.1)​ |
| Time on Treatment​ | -​ | 2.6 [0.7, 7.4]​ | 1.4 [0.5, 4.1]​ |
| Glucocorticoids (%)​ | -​ | 62 (64.6)​ | 28 (80)​ |
| csDMARD​ (%)​ | -​ | 57 (59.4)​ | 18 (51.4)​ |
| Biologic (%)​ | -​ | 7 (7.3)​ | 1 (2.8)​ |

*Data is median [IQR] or N (%) where indicated. *For Takayasu Arteritis patients (TAK), active disease: ITAS2010 score ≥ 1 or ITAS.CRP ≥ 2; for large vessel giant cell arteritis patients (LV-GCA): NIH score ≥ 2 at the time of sampling. CRP, C-reactive protein; ESR, erythrocyte sedimentation rate. Treatment at time of sampling is summarised. csDMARD, conventional synthetic disease modifying anti-rheumatic drug; Biologic, targeted biologic agent e.g. anti-IL6R monoclonal antibodies or TNF inhibitor.*

**Supplementary Table 2: C-GCA cohort**

|  | **Not C-GCA** | **C-GCA** |
| --- | --- | --- |
| N | 89 | 150 |
| Age | 69 [62, 76] | 73.5 [67, 78] |
| Female (%) | 63 (70.8) | 108 (72) |
| White European^Φ^ (%) | 88 (98.9) | 149 (99.3) |
| TAB+ (%)* | 0 (0) | 56 (37.3) |
| USS Positive (%) | 27 (30.3) | 78 (52) |
| Cranial Ischaemic Complications (%) | 18 (20.2) | 33 (22) |
| Polymyalgic symptoms (%) | 7 (7.9) | 18 (12) |
| ESR, mm/hr*ꭞ* | 13 [5, 28] | 35 [19.5, 58] |
| CRP, mg/L*ꭞ* | 14 [4, 22.5] | 30 [12.7, 54] |
| Platelets, 10^9^/L*ꭞ* | 290 [236, 332] | 348 [277, 454] |

*Data is median [IQR] or N (%) where indicated.* ^Φ^ Ethnicity was self-identifed. **Six patients did not have TAB result available. ꭞOnly partial data available: Not C-GCA N = 81, 55, 89 for ESR, CRP and platelets respectively; C-GCA N = 143, 98, 147. TAB, temporal artery biopsy; USS, temporal artery ultrasound sonography; Cranial Ischaemic Complications (as per methods); ESR, erythrocyte sedimentation rate; CRP, C-reactive protein.*

## Supplementary Table 3. Performance of predictive models for discrimination of biopsy-proven C-GCA from Not C-GCA

|  | **11-protein LASSO** | **CXCL9**  **LR univariate** | **DPP4**  **LR**  **univariate** | **SERPINA5**  **LR**  **univariate** | **IL6**  **LR**  **univariate** |
| --- | --- | --- | --- | --- | --- |
| Accuracy  (95% CI) | 0.87  (0.73, 0.96) | 0.85  (0.69, 0.94) | 0.82  (0.66, 0.92) | 0.79  (0.64, 0.91) | 0.69  (0.52, 0.83) |
| P-Value [Acc > NIR] | 0.0004 | 0.0016 | 0.005 | 0.014 | 0.20 |
| Kappa | 0.73 | 0.67 | 0.63 | 0.54 | 0.28 |
| Sensitivity | 0.80 | 0.73 | 0.80 | 0.60 | 0.33 |
| Specificity | 0.92 | 0.91 | 0.83 | 0.92 | 0.92 |
| Pos Pred Value | 0.86 | 0.84 | 0.75 | 0.82 | 0.71 |
| Neg Pred Value | 0.88 | 0.84 | 0.87 | 0.79 | 0.69 |

*Metrics of model performance were calculated in the Test set samples, which had not been previously seen by the model. Shown here are the 11-protein multivariate model (LASSO), and univariate logistic regression (LR) models for the best three markers (CXCL9, DPP4 and SERPINA5), and IL6. CI = confidence interval. P-Value represents the p-value for a one-sided exact binomial test of whether the accuracy (Acc) is better than the ‘no information rate’ (NIR), defined as the largest class percentage in the Test set (here 0.615). Metrics are rounded to 2 significant figures.*

## Supplementary Table 4: Confusion matrix comparing model predictions from the 11-protein LASSO model in the Test set versus true labels


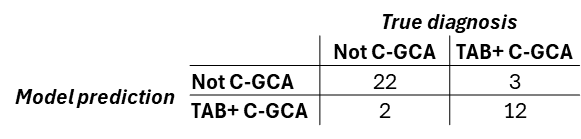


# **Supplementary Figures**


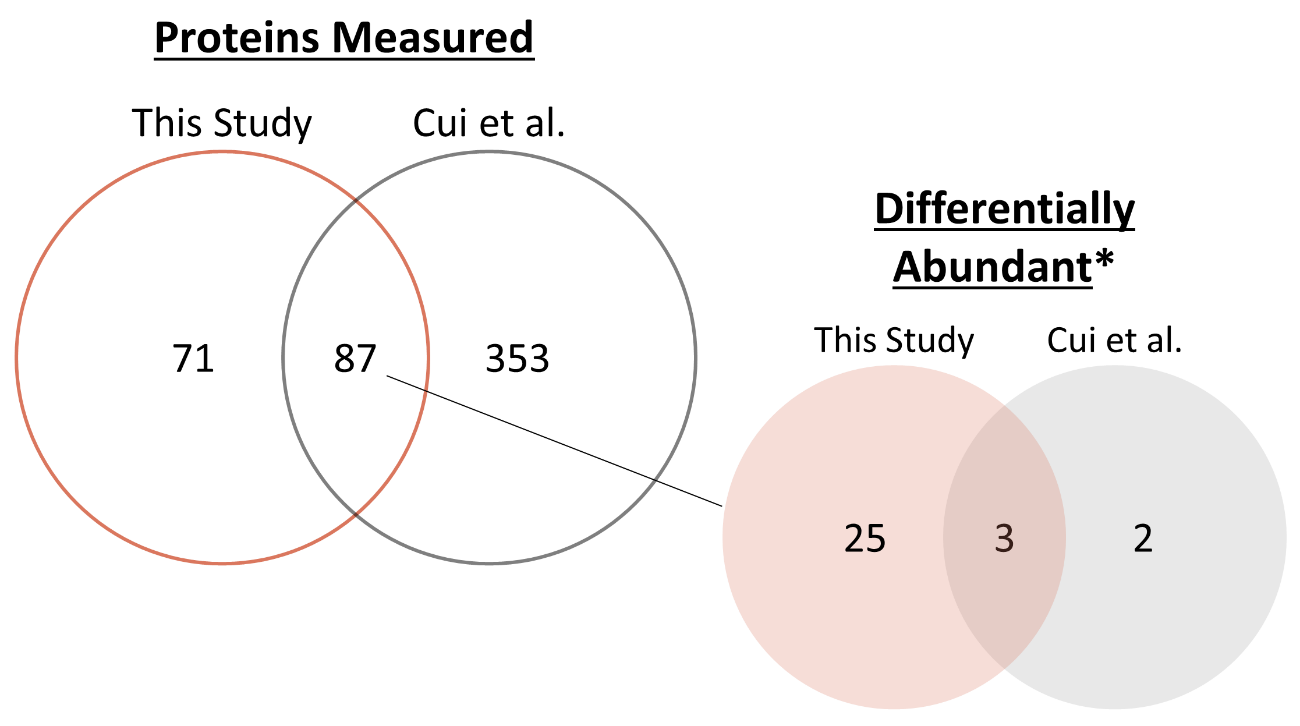


## Figure S1. Comparing TAK findings to those of a previous plasma proteomic study

*The results of the Takayasu Arteritis (TAK) vs healthy control analysis were compared to those of the largest comparable plasma proteomic study*^1^*. 87 proteins were common between the 158 proteins that passed quality control checks in our analysis and the 440 proteins measured in the previous study (Venn diagram on LHS). Within the subset of proteins measured in both studies, 3 were identified as differentially abundant in both studies (Venn Diagram RHS). In this study, *differential abundance was defined as Benjamini-Hochberg Adjusted P < 0.05 while in study of Cui et al, the published definition of significance was used.*

##
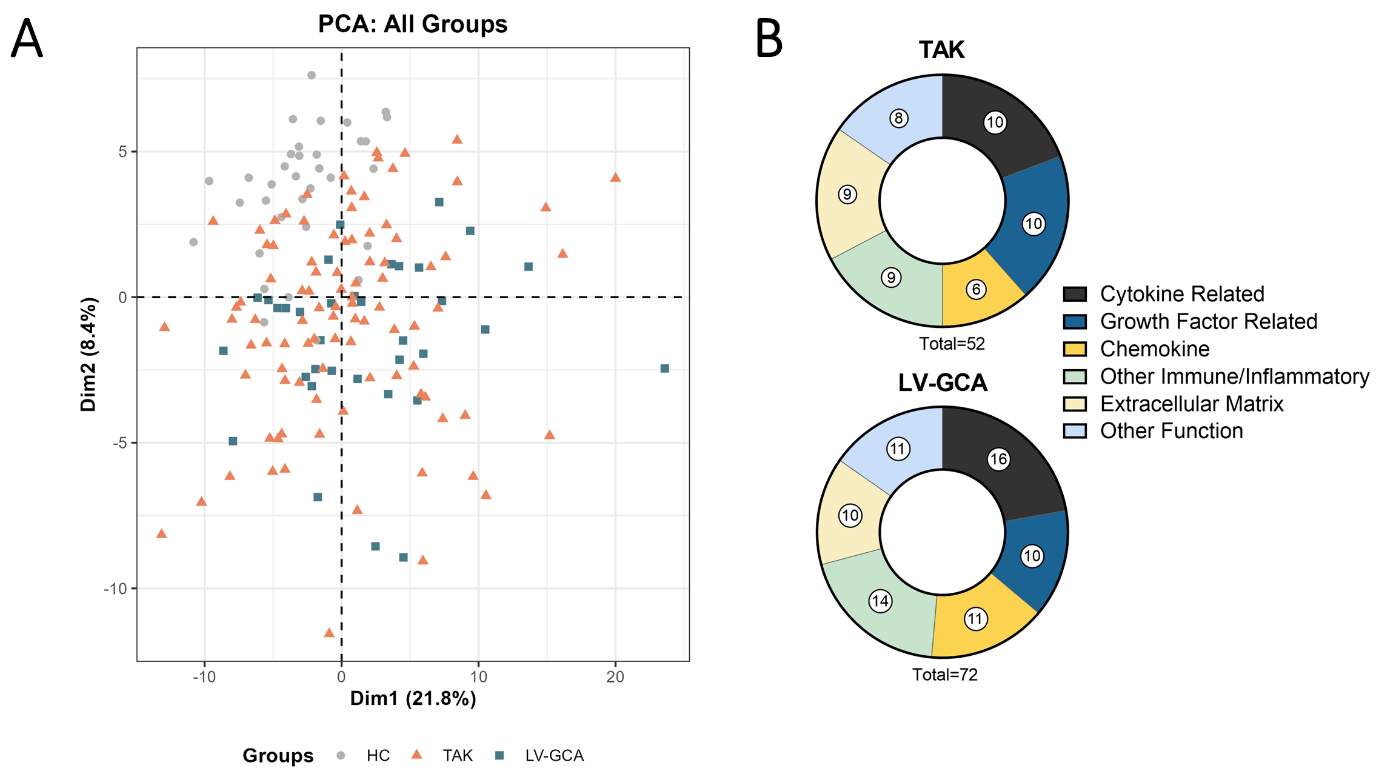
Figure S2. TAK and LV-GCA vs HC comparison

*A) Principal component analysis of the scaled relative abundance values of 158 proteins in Takayasu arteritis patients (TAK, N=94), large vessel giant cell arteritis patients (LV-GCA, N=35) and healthy control participants (HC, N=34). Only subjects with complete proteomic data could be included. B) Functional categories of differentially abundant proteins in TAK vs HC and LV-GCA vs HC comparisons.*

##
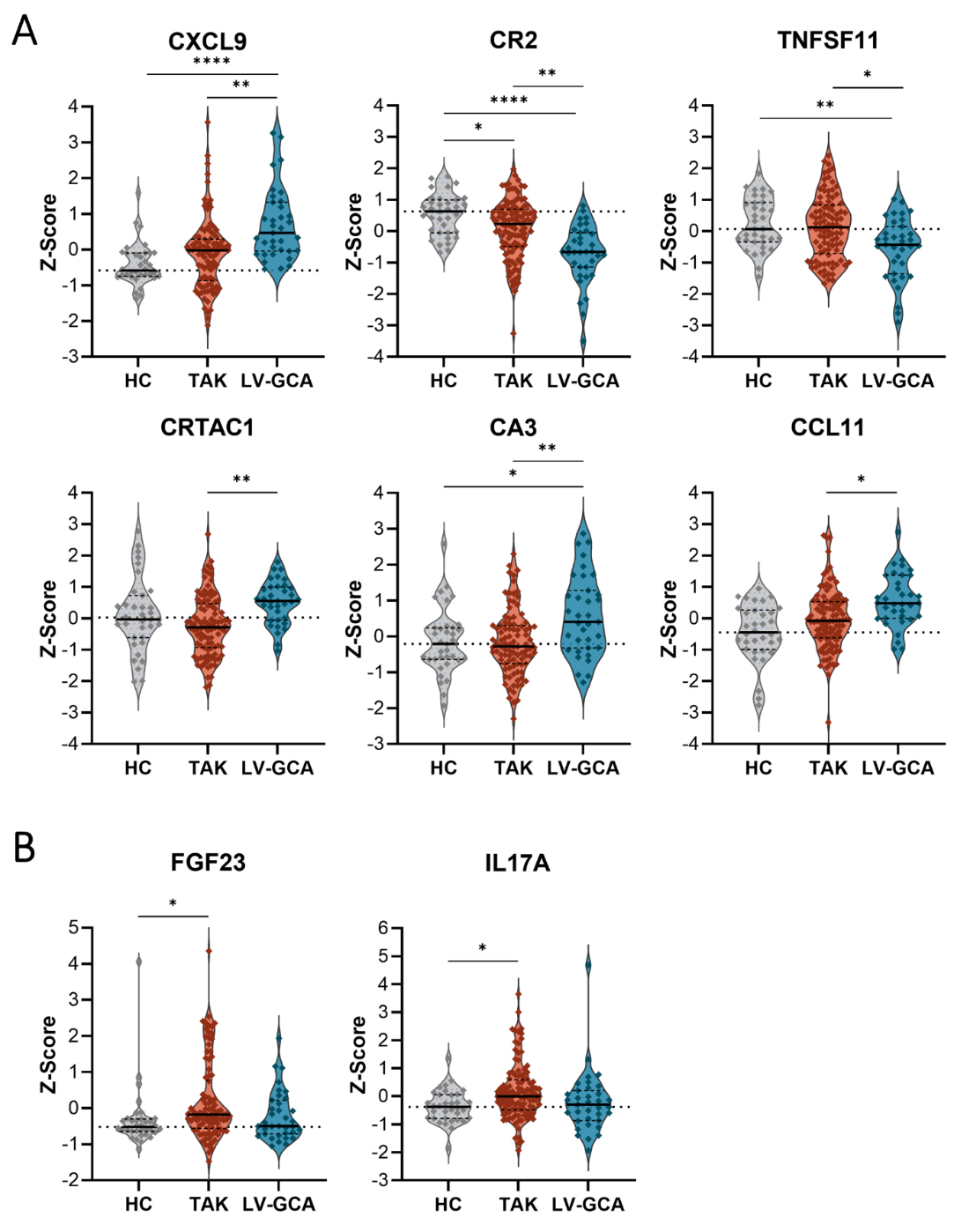
Figure S3. Plasma protein differences between TAK and LV-GCA patients

*A) Violin plots showing scaled relative abundance values (Z-Score) of differentially abundant proteins between Takayasu arteritis patients (TAK, N=96) and large vessel giant cell arteritis patients (LV-GCA, N=35). B) Violin plots of proteins with most notable TAK-specific differences compared to LV-GCA patients. Relative abundance values of healthy control participants (HC) also shown for reference.* *Benjamini-Hochberg adjusted P < 0.05: *, P ≤ 0.01: **, P ≤ 0.001: ***, P ≤ 0.0001: ****.*


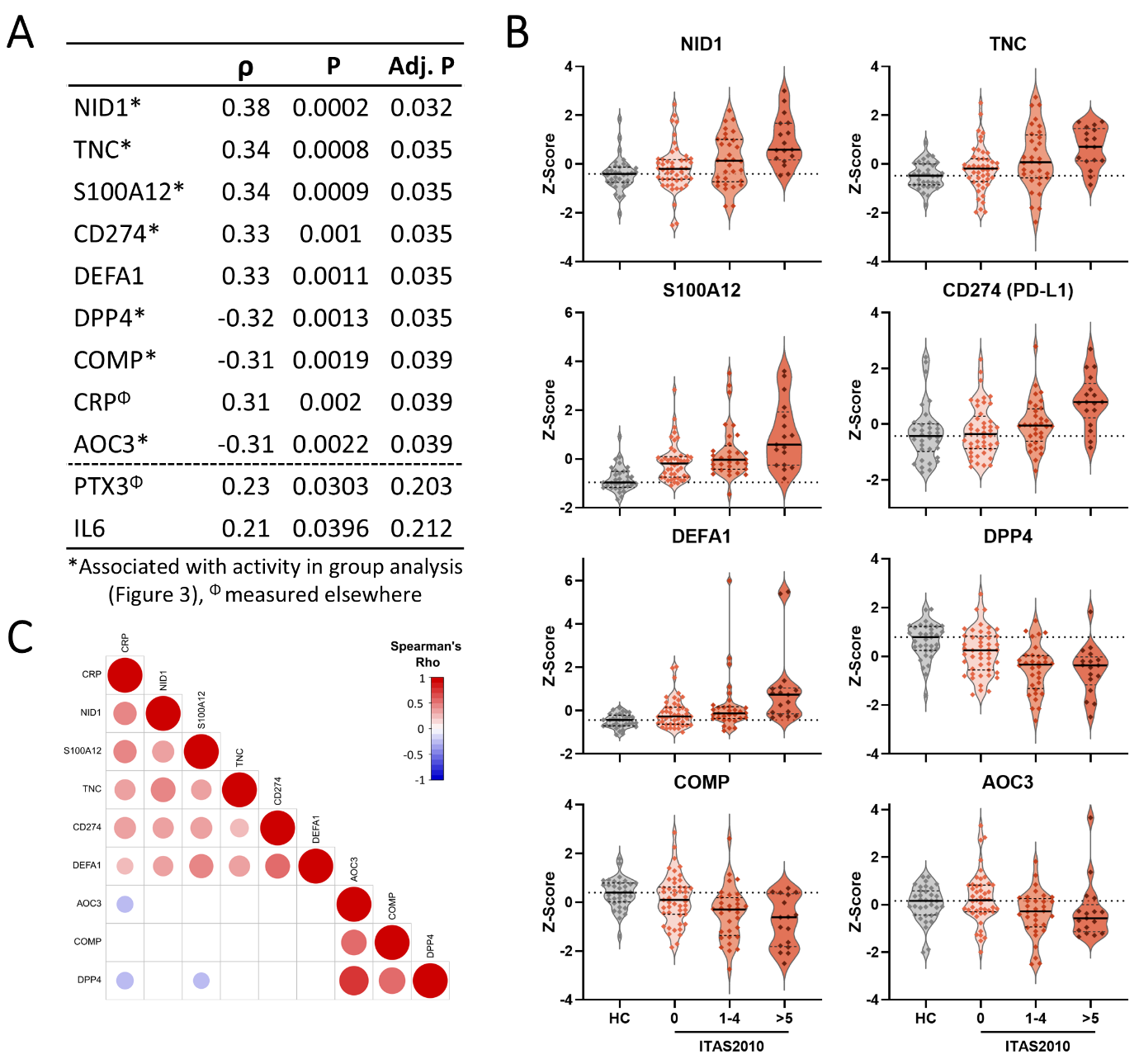


## Figure S4. Proteins associated with Disease Activity Score in TAK

*A) The Spearman correlation of each protein (N=158) with the Indian Takayasu Clinical Activity Score (ITAS2010) disease activity score was tested in Takayasu Arteritis patients (N=96), proteins with significant associations (Benjamini-Hochberg Adjusted P < 0.05) shown. Spearman rho correlation coefficients are shown (ρ). ITAS2010 correlation with C reactive protein (CRP) and pentraxin-3 (PTX3), measured separately, are also shown for comparison. B) Violin plots showing scaled relative abundance values (Z-score) of disease activity associated proteins in TAK patients stratified by ITAS2010 range (orange groups, range indicated on X axis). Z-scores in healthy controls are also shown for comparison (grey). C) Matrix showing significant (unadjusted P < 0.05) spearman correlations between disease activity associated proteins and CRP, circle size and colour intensity are proportional to Spearman’s Rho value as per legend.*

**
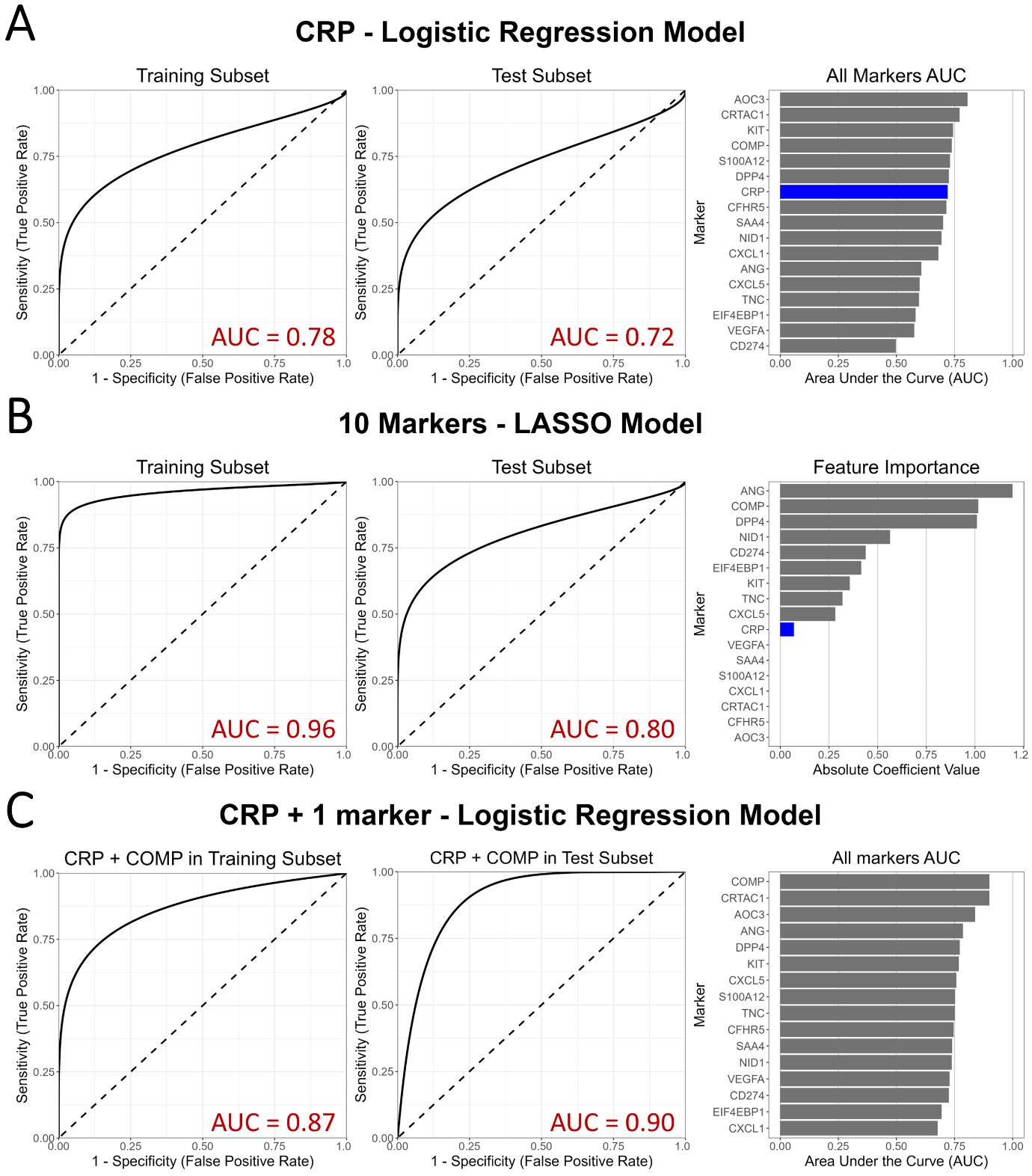
**

## Figure S5. Novel markers provide improved detection of disease activity compared to CRP alone in TAK

*A) Receiver operating characteristic (ROC) curves showing the performance of the CRP univariate logistic regression model in discriminating active from inactive Takayasu arteritis. LHS, performance in Training subset (N=71); Middle, performance in Test subset (N=23); RHS, barplot showing area under the curve (AUC) values corresponding to univariate model performance of each marker in Test subset. B) ROC curves showing the performance of multivariate LASSO model in Test and Train subsets. The barplot shows the model coefficients in absolute values for each marker; 10 markers were selected and weighted by the LASSO model. C) ROC curves showing the performance of CRP + COMP in Test and Train subsets. The barplot shows the AUC values for each 2 marker model (CRP + additional marker) in the Test subset; CRP + COMP had the highest value.*


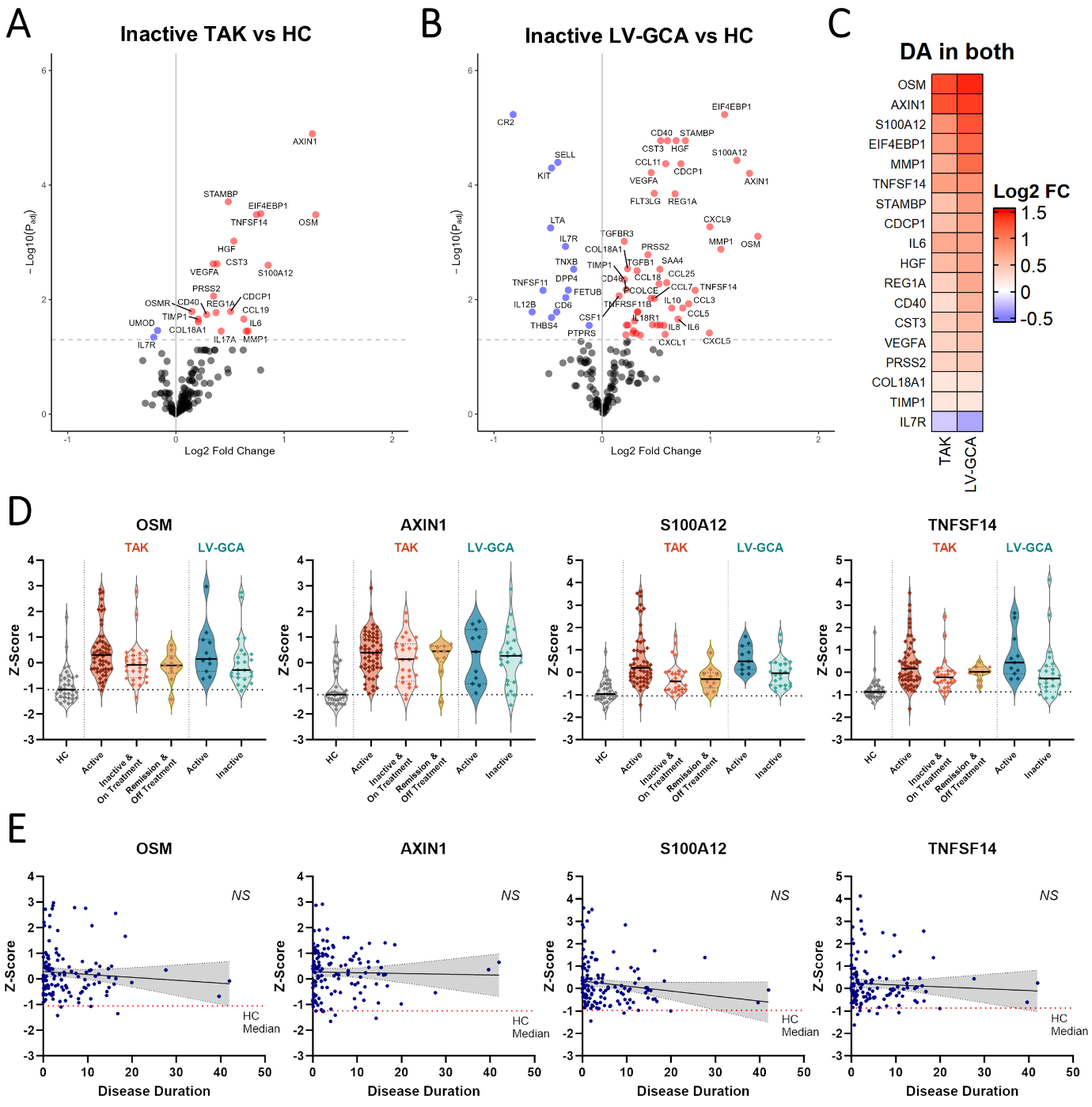


## Figure S6. Differentially abundant proteins in inactive TAK & LV-GCA

*Volcano plots showing results of differential abundance analyses: A) Takayasu Arteritis (TAK) patients with inactive disease (N=40) vs healthy control participants (HC, N=35) B) Large Vessel Giant Cell Arteritis (LV-GCA) patients with inactive disease (N=24) vs HC. -Log10(P_adj_) = -Log_10_ transformed Benjamini-Hochberg adjusted p-value. Red and blue indicate proteins that are significantly (P_adj_ < 0.05) upregulated and downregulated, respectively. C) Heatmap showing Log_2_ fold changes of the 18 proteins that were significantly altered in both inactive TAK and LV-GCA. D) Violin plots showing scaled abundance values (Z-Score) of proteins with prominent elevations in both inactive TAK and LV-GCA (P_adj_ < 0.05) according to active and inactive status (X axis). Inactive TAK patients are further separated into two groups: i) clinically inactive (ITAS2010=0) but on treatment (Pink, N=30) and ii) durable clinical remission, achieving cessation of all immunosuppressive treatments (Yellow, N=10). E) Scatterplot showing Z-score of elevated proteins vs disease duration where red dashed line shows median of HCs for same protein. Association of Z-scores with disease duration was tested using linear regression (all were insignificant P > 0.05).*

**
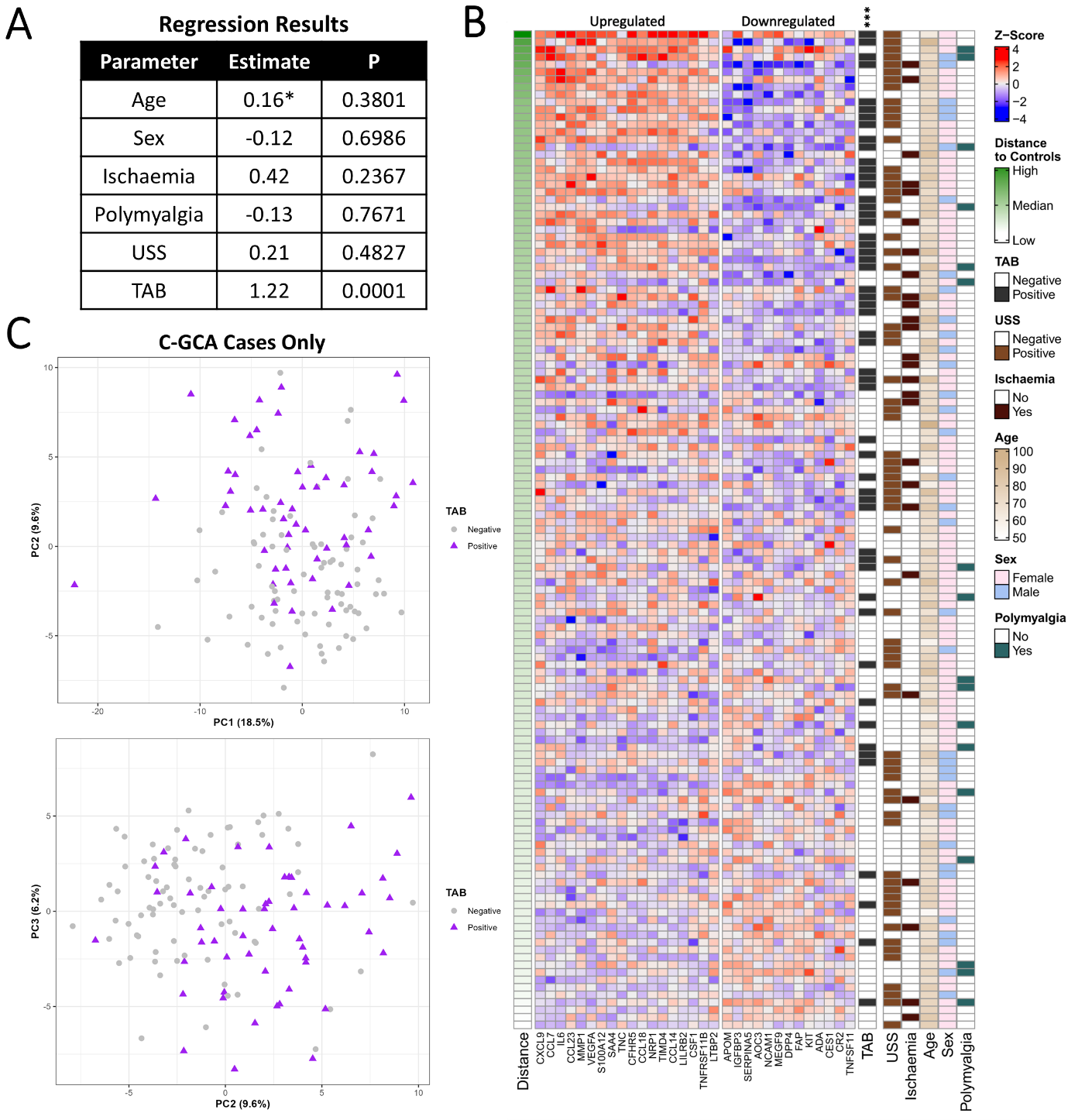
**

## Figure S7. Proteomic changes in C-GCA are most pronounced in biopsy proven disease

*There were 31 differentially abundant proteins (DAPs) identified in the comparison of cranial giant cell arteritis patients (C-GCA) and Not C-GCA cases. Euclidean distance of each C-GCA patient to the Not C-GCA mean level for the 31 DAPs was calculated (as a change score) and its association with age, sex, cranial ischaemic complications (ischaemia), polymyalgic symptoms (polymyalgia), temporal artery ultrasound sonography (USS) and biopsy (TAB) results was tested by multiple linear regression. A) Results of linear regression. Estimate for age is that of a continuous variable (*) with the other parameters being categorical. B) Heatmap depicting the association between Euclidean distance (LHS), scaled relative abundance of DAPs (Z-scores) and clinical parameters (RHS) in C-GCA patients (rows). C) Principal component analysis of the scaled relative abundance values of 167 proteins in biopsy proven C-GCA patients (purple points) and biopsy negative C-GCA patients (grey points). 1^st^ vs 2^nd^ principal components (PC1 vs PC2) and PC2 vs PC3 are shown.*


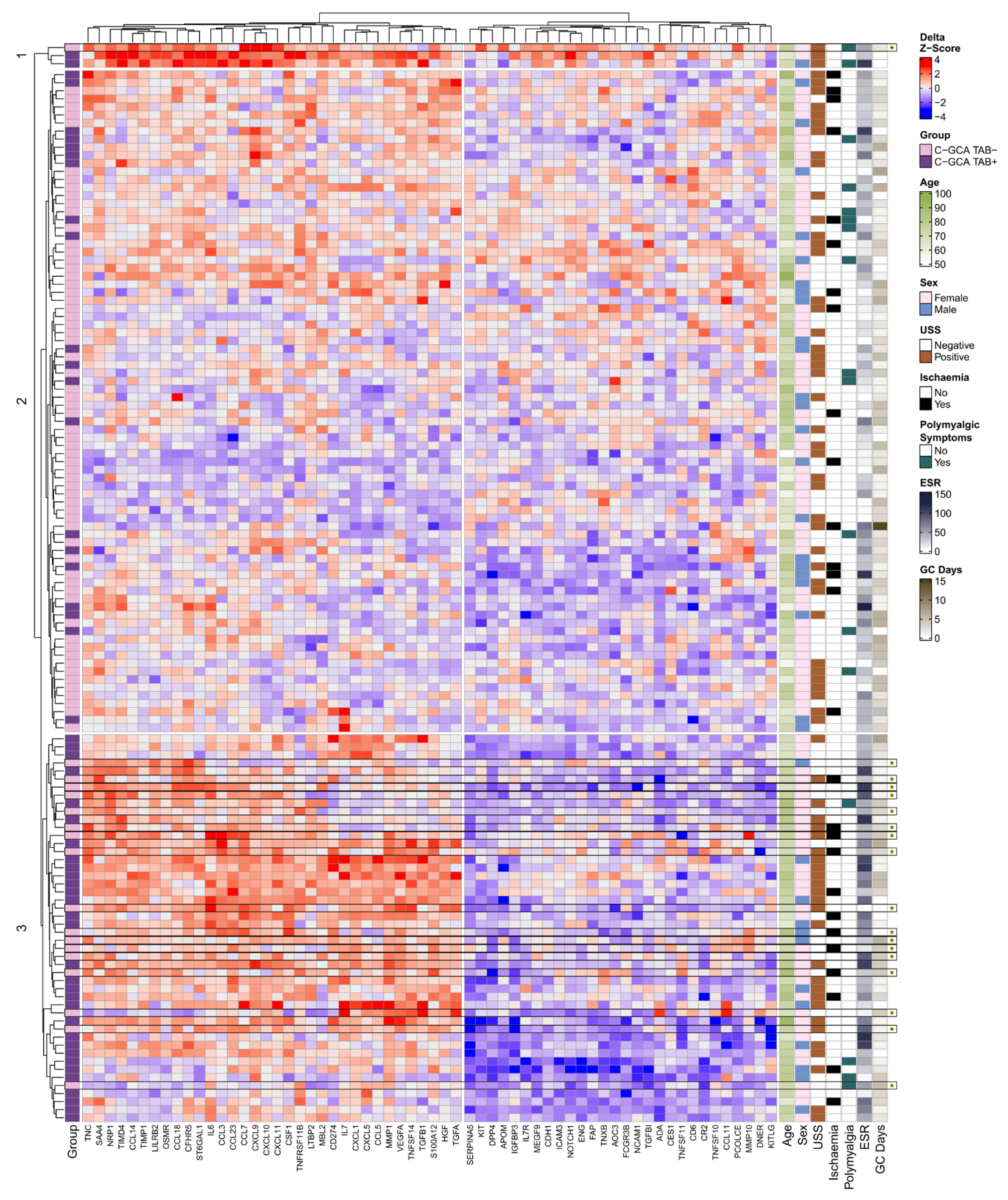


## Figure S8. Comparison of proteomic changes in TAB+ and TAB- C-GCA patients

*62 proteins were identified as differentially abundant in temporal artery biopsy positive (TAB+) cranial giant cell arteritis patients (C-GCA) vs Not C-GCA cases. Heatmap depicts the change in abundance of these proteins in individual C-GCA patients compared to Not C-GCA mean levels (Delta Z-Score). C-GCA patients are annotated as TAB- (pink) or TAB+ (purple) on LHS.* *Clinical parameters shown on RHS including age, sex, temporal artery ultrasound sonography (USS) result, cranial ischaemic complications (Ischaemia), polymyalgic symptoms, erythrocyte sedimentation rate (ESR) and days on glucocorticoid treatment. The majority of TAB- C-GCA patients formed a single cluster (#2) with only minor proteomic changes. 18 TAB- patients (22.5%) had statistically similar changes to TAB+ patients (marked with box and * on RHS) but this pattern was unrelated to clinical parameters. Only C-GCA cases with complete proteomic data and TAB result available were included (N=133).*

## *
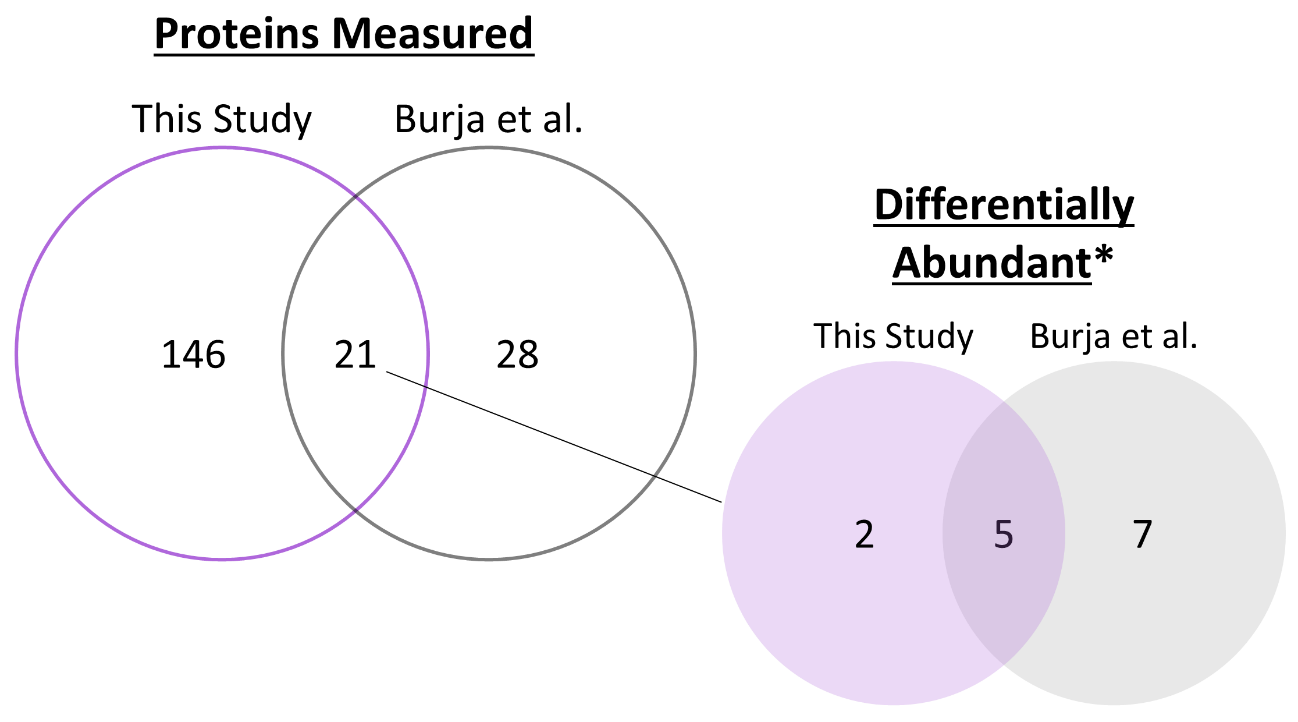
*Figure S9. Comparing biopsy proven C-GCA findings to those of a previous plasma proteomic study

*Differential abundance results of biopsy proven cranial giant cell arteritis (C-GCA TAB+) vs Not C-GCA cases were compared to those of the largest comparable plasma proteomic study*^2^*. 21 proteins were common between the 167 proteins that passed quality control checks in our analysis and the 49 proteins measured in the previous study (Venn diagram on LHS). Within the subset of proteins measured in both studies, 5 were identified as differentially abundant in both studies (Venn Diagram RHS). In this study, *differential abundance was defined as Benjamini-Hochberg Adjusted P < 0.05 while in study of Burja et al, the published definition of significance was used.*


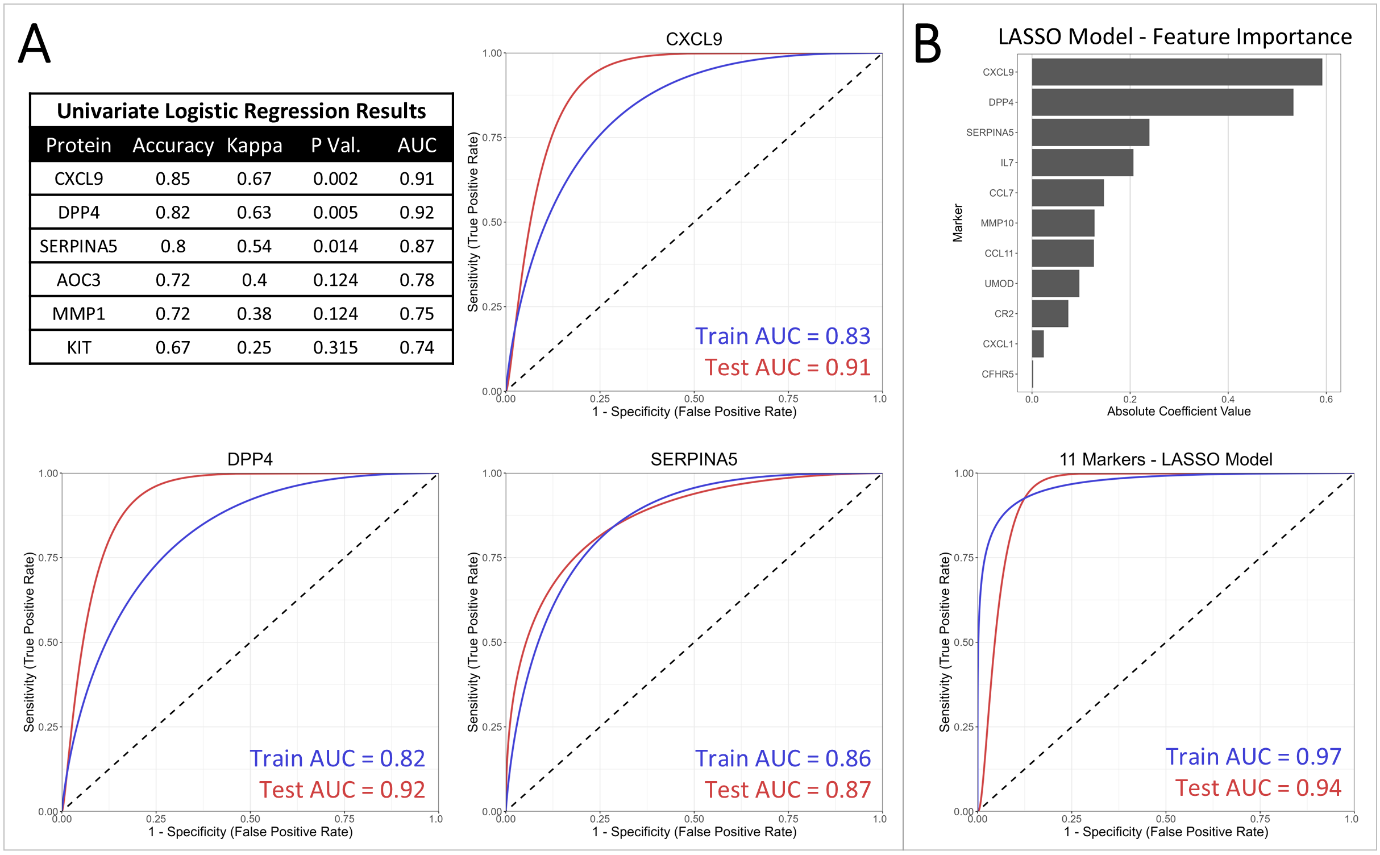


## Figure S10. Supervised learning to identify diagnostic markers of biopsy-proven C-GCA

*A) Results of univariate logistic regression models for diagnosis of cranial giant cell arteritis (C-GCA). Table (upper left): accuracy, kappa and area under the curve (AUC) values for the performance of each protein model in the Test set. P Val represents the p-value for a one-sided binomial exact test of whether the accuracy is better than the "no information rate," defined as the largest class percentage in the Test set. Receiver operating characteristic (ROC) curves for the three proteins with the best univariate model performance (CXCL9, DPP4 and SERPINA5). B) Results of multivariate LASSO model for diagnosis of C-GCA. The barplot shows the absolute (i.e. unsigned) model coefficients for the 11 markers selected by the model. ROC curve shows the performance of the 11-protein LASSO model in the Test and Train subsets. AUC values are indicated on the graph.*

**
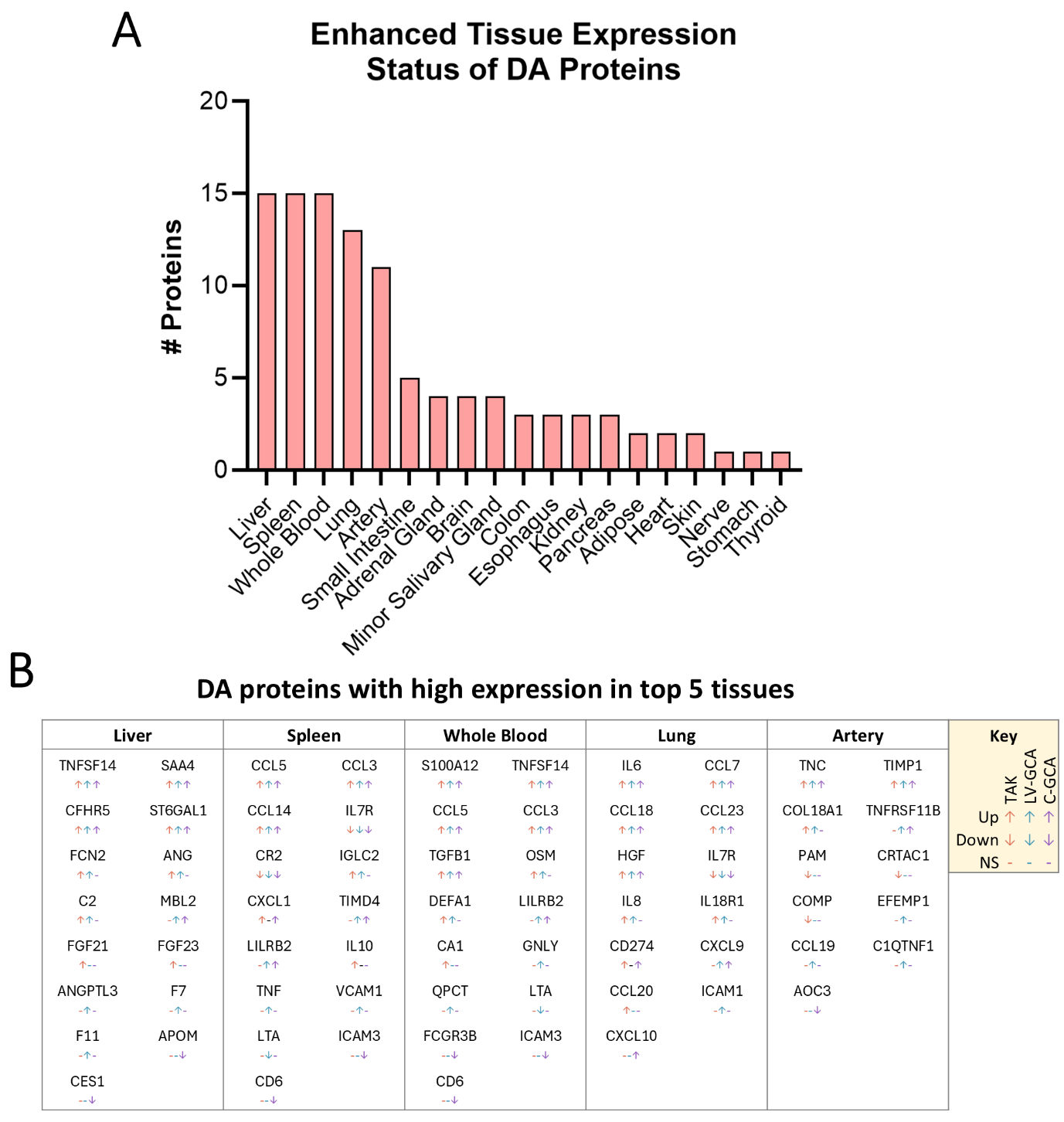
**

## Figure S11. Tissue expression of LVV-associated plasma proteins

*The tissue expression profile of proteins identified as differentially abundant (DA) in active Takayasu arteritis (TAK), active large vessel giant cell arteritis (LV-GCA) or biopsy proven cranial GCA (C-GCA) was investigated using the GTEx bulk RNA-seq database*^7^*. Proteins with enhanced (or high) tissue expression were defined as having >4 fold higher than the average expression of other tissues. A) Barplot showing number of proteins with enhanced levels per tissue type. B) Table showing proteins identified as enhanced in top 5 represented tissues. Arrows under protein names denote significant upregulation (↑), downregulation (↓) or no change (-) per large vessel vasculitis (LVV) type as per key (RHS).*

**
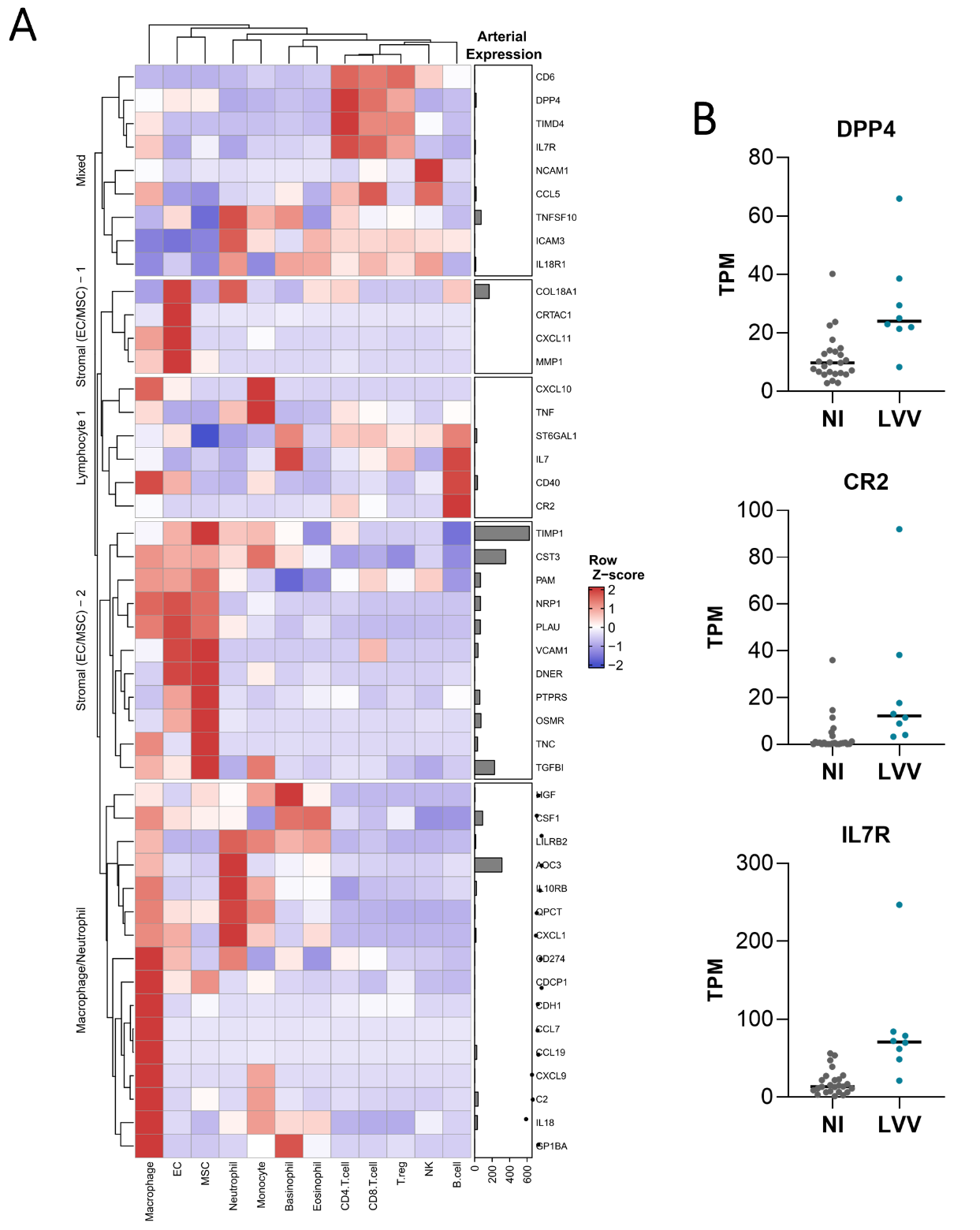
**

## Figure S12. Cell-type expression profile of proteins/genes identified as dysregulated in both LVV plasma and tissue

*47 proteins/genes had significantly altered levels in both large vessel vasculitis (LVV) plasma and in aortic tissue affected by LVV. A) Heatmap depicting the expression profile of these proteins in 10 immune and 2 non-immune cell types from the Blueprint bulk RNA-seq database*^6^*. One protein/gene (PRSS2) is not shown due to negligible expression. Clusters produced using Euclidean distance and complete linkage were manually annotated. Arterial expression from GTEx database*^7^ *is shown on RHS in transcript per million values (TPM). B) Dot plots showing TPM values for proteins that were downregulated in the plasma of patients with all 3 LVV types but upregulated in arterial tissue affected by LVV. EC, endothelial cell; MSC, mesenchymal stem cell; NK, natural killer cell; T.reg regulatory T-cell. Full cell definitions and isolation methods as per Blueprint protocols.*

# **References**

1. Cui X, Qin F, Song L, Wang T, Geng B, Zhang W, et al. Novel Biomarkers for the Precisive Diagnosis and Activity Classification of Takayasu Arteritis. Circ Genomic Precis Med. 2019 Jan;12(1):e002080.

2. Burja B, Feichtinger J, Lakota K, Thallinger GG, Sodin-Semrl S, Kuret T, et al. Utility of serological biomarkers for giant cell arteritis in a large cohort of treatment-naïve patients. Clin Rheumatol. 2019 Feb 1;38(2):317–29.

3. Tombetti E, Hysa E, Mason JC, Cimmino MA, Camellino D. Blood Biomarkers for Monitoring and Prognosis of Large Vessel Vasculitides. Curr Rheumatol Rep. 2021 Feb 10;23(3):17.

4. Kuhn M. Building Predictive Models in R Using the caret Package. J Stat Softw. 2008 Nov 10;28:1–26.

5. Friedman JH, Hastie T, Tibshirani R. Regularization Paths for Generalized Linear Models via Coordinate Descent. J Stat Softw. 2010 Feb 2;33:1–22.

6. Fernández JM, de la Torre V, Richardson D, Royo R, Puiggròs M, Moncunill V, et al. The BLUEPRINT Data Analysis Portal. Cell Syst. 2016 Nov 23;3(5):491-495.e5.

7. Lonsdale J, Thomas J, Salvatore M, Phillips R, Lo E, Shad S, et al. The Genotype-Tissue Expression (GTEx) project. Nat Genet. 2013 Jun;45(6):580–5.
